# Thin film design of amorphous hafnium oxide nanocomposites enabling strong interfacial resistive switching uniformity

**DOI:** 10.1126/sciadv.adg1946

**Published:** 2023-06-21

**Authors:** Markus Hellenbrand, Babak Bakhit, Hongyi Dou, Ming Xiao, Megan O. Hill, Zhuotong Sun, Adnan Mehonic, Aiping Chen, Quanxi Jia, Haiyan Wang, Judith L. MacManus-Driscoll

**Affiliations:** ^1^ Department of Materials Science & Metallurgy, University of Cambridge, 27 Charles Babbage Rd, Cambridge CB3 0FS, UK.; ^2^ Department of Engineering, University of Cambridge, 9 JJ Thompson Avenue, Cambridge CB3 0FA, UK.; ^3^ Department of Physics, Linköping University Thin Film Physics Division, 581 83 Linköping, Sweden.; ^4^ School of Materials Engineering, Purdue University, Neil Armstrong Hall of Engineering, 701 West Stadium Avenue, West Lafayette, IN 47907-2045, USA.; ^5^ Department of Electronic & Electrical Engineering, University College London, London WC1E 7JE, UK.; ^6^ Center for Integrated Nanotechnologies (CINT), Los Alamos National Laboratory, Los Alamos, NM 87545, USA.; ^7^ Department of Materials Design and Innovation, University at Buffalo, 136 Bell Hall, Buffalo, NY 14260, USA.

## Abstract

A design concept of phase-separated amorphous nanocomposite thin films is presented that realizes interfacial resistive switching (RS) in hafnium-oxide-based devices. The films are formed by incorporating an average of 7% Ba into hafnium oxide during pulsed laser deposition at temperatures ≤400°C. The added Ba prevents the films from crystallizing and leads to ∼20-nm-thin films consisting of an amorphous HfO*_x_* host matrix interspersed with ∼2-nm-wide, ∼5-to-10-nm-pitch Ba-rich amorphous nanocolumns penetrating approximately two-thirds through the films. This restricts the RS to an interfacial Schottky-like energy barrier whose magnitude is tuned by ionic migration under an applied electric field. Resulting devices achieve stable cycle-to-cycle, device-to-device, and sample-to-sample reproducibility with a measured switching endurance of ≥10^4^ cycles for a memory window ≥10 at switching voltages of ±2 V. Each device can be set to multiple intermediate resistance states, which enables synaptic spike-timing–dependent plasticity. The presented concept unlocks additional design variables for RS devices.

## Teaser

Highly promising performance for future computing applications is achieved based on a new materials design.

## INTRODUCTION

Owing to the simplicity of fabrication and complementary metal-oxide semiconductor (CMOS) compatibility, resistive switching (RS) devices are among the prime contenders for future energy-efficient memory and computing technologies such as neuromorphic or in-memory computing (*1*–*3*). RS in a device refers to its ability to exhibit different controlled electrical resistance states between its electrical contacts. For large-scale industrial integration, such RS functionality needs to be realized with materials that are well established in industry, and one such material is amorphous hafnium oxide, which is widely used as the gate oxide in semiconductor field-effect transistors. While there have been numerous demonstrations of RS in hafnium-oxide-based devices, the vast majority of those relied on the reversible dielectric breakdown of the devices, i.e., the reversible formation of a conductive filament through the otherwise insulating film (*4*–*8*). In an amorphous material, the formation of such a filament is inherently stochastic, and typically, it is accompanied by very abrupt changes in the device current. This switching stochasticity induces inherent variability both in terms of cycle-to-cycle repeatability within the same device and in terms of device-to-device consistency, which in turn undermine reliable circuit integration (*9*, *10*). In addition, often an initial forming step is required with a higher voltage than for the subsequent device operation and with an external current compliance, to create the very first filaments (*11*).

So far, demonstrations of gradual/interfacial RS in amorphous hafnium oxide required an additional layer in the oxide stack such as substoichiometric AlO*_x_* (*12*), TiO*_x_* (*13*), or WO*_x_* (*14*), and/or data on endurance and/or retention were missing or not reported in detail (*12*, *13*, *15*). In addition, even if the observed RS is gradual, often it is not entirely clear whether the switching is interface-controlled or whether it is effectively filamentary switching with a current-limiting series resistance due to the added layers. Reports of interfacial/gradual RS in many other materials systems were based on complex perovskites (*16*), which are not industry-compatible, or they relied on the incorporation of other industry-incompatible materials in otherwise compatible oxides, such as Pt nanoparticles dispersed in SiO_2_ (*17*). Compared with other industry-compatible oxides where gradual/interfacial RS has been demonstrated, for example TiO*_x_* (*18*) or TaO*_x_* (*19*), hafnium oxide offers the additional versatility of having ferroelectric (FE) (crystalline) phases (*20*), which may provide an additional edge among the competition for future memory and neuromorphic devices. Furthermore, the focus of many reports is often on individual device demonstrations, but it is important to achieve and report uniform performance beyond single devices (*21*).

Here, we demonstrate uniform and stable cycle-to-cycle, device-to-device, and sample-to-sample repeatability in interfacial RS devices based on only one deposited hafnium oxide layer. To achieve this, we designed a self-assembled amorphous nanocomposite thin film, deposited at CMOS-compatible temperatures ≤400°C, which consists of a parent amorphous hafnium-oxide-based matrix and nanoscale columnar second-phase regions to support the RS process. To create this amorphous nanocomposite, we added Ba to hafnium oxide during the single-step thin film deposition, and because of its simplicity of compositional control, we used pulsed laser deposition (PLD) to deposit the films.The PLD target had a Ba:Hf cation ratio of 1:2, which exceeds the solubility limit of dopants (with large atomic radii) in (crystalline) hafnium oxide, so that the formation of a second (amorphous) phase could be expected (*22*). For future work, it will be worthwhile to transfer the insights gained from the present study to, e.g., a sputter deposition process for increased industry compatibility.

The incorporation of Ba in the hafnium oxide thin films induces three key effects that produce high-performance interfacial RS in the films: (i) It leads to materials amorphization, which causes material uniformity on a microscopic level, more so than any pure PLD hafnium oxide deposited over a wide range of temperatures. (ii) It reduces the relative oxygen content of the films and the Hf oxidation states, which makes the films more electronically conducting and thus prevents the build-up of large electric fields over the films, which would lead to dielectric breakdown. (iii) It produces second-phase nanocolumnar structures in the thin films, which facilitate the controlled forming of only partial filaments without a complete dielectric breakdown, and the corresponding forming voltage of 2 V is the same as the subsequent maximum (positive) switching voltage. These columns extend from the top electrode (TE) of the devices, where they short-circuit any Schottky-like barrier, and because they reach about two-thirds through the film thickness, they restrict the RS process to the Schottky-like barrier at the bottom interface. As a result, the Ba:HfO*_x_* nanocomposite films exhibit uniform RS with low variability and low switching voltages of ±2 V, and without the abrupt current changes characteristic of filamentary switching that predominates for RS in standard single-layer amorphous hafnium oxide thin films. To date, we are not aware of any demonstration of similarly stable and repeatable interface-controlled RS in single-film hafnium-oxide-based devices.

Besides the realization of interface RS in hafnium oxide, this demonstration of amorphous phase-separated nanocomposite thin films is momentous because so far, for RS applications, the vast majority of composite thin films consisted either of phase-separated epitaxial composites [e.g., (*23*)] or of homogeneously mixed amorphous or crystalline films such as the widely studied (ferroelectric) doped hafnium oxide (*20*). As demonstrated in the following, phase-separated amorphous oxide nanocomposites have the potential to add an additional tuneable dimension to the design of thin film functionality, and it should be possible to extend the concept to other industrially important materials systems such as SiO*_x_*.

## RESULTS

We first present and discuss the electrical performance of the devices including neuromorphic functionality and then present the materials analysis data. On this basis, we propose an explanation for the switching and conduction mechanisms.

### Devices and electrical performance

**Fig. 1.**
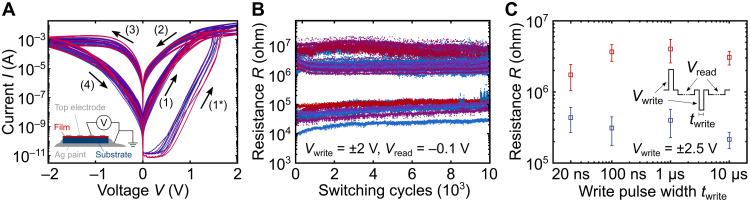
*I*-*V* curves, switching endurance, and switching speed measurements. (**A**) Initial five *I*-*V* curves of 10 different pristine amorphous nanocomposite devices with Ba:HfO*_x_* deposited at 400°C. Each device is shown in a different color. TE diameters were 100 and 50 μm. A clear forming process (1*) is observed during the first voltage application, but it does not require a higher voltage than the subsequent switching. The switching sequence is indicated by numbered arrows and the curves of all devices overlap each other closely. Inset: Schematic of device and measurement geometry for devices with Nb:STO BEs. (**B**) Endurance of 10^4^ switching cycles with a memory window of ≥10 for 10 different devices from two different samples. Thin films for both deposited at 400°C, different color for each device. (Endurance figure with logarithmic *x* axis in fig. S1F.) (**C**) Measurements of device switching speed. Each data point is the mean value of 20 switching iterations, and the error bars represent the measurement SD. Fast measurements with ±2 V in figs. S7 and S8. Inset: Switching sequence for endurance and switching speed measurements. For endurance (B), *t*_write_ = *t*_read_ ≈ 22 ms; for switching speed (C), *t*_write_ is displayed on the *x* axis and *t*_read_ was a few ms.

Ba:HfO*_x_* thin films were deposited directly on electrically conductive single-crystal (001)-oriented Nb-doped strontium titanate (Nb:STO) substrates with a supplier-provided resistivity ρ ≈ 5.5 milliohm·cm at 300 K. Nb:STO was chosen as the initial substrate to study our thin film design concept with a ‘clean’ (conductive, no native oxides, minimal surface roughness) reference bottom electrode (BE). While a commercial process for the integration of STO on 200-mm Si wafers has been demonstrated (*24*), Nb:STO is typically not regarded as an industry-compatible electrode. Thus, in fig. S3C, we also present initial data of our films deposited on TiN. The thin films were deposited by PLD from an oxide ceramic target consisting of HfO_2_ and BaHfO_3_ in a molar ratio of 1:1. TiN was deposited by PLD as well. While its use is increasing, both in terms of the number of users and in terms of substrate sizes, similar to the argument about Nb:STO, PLD is typically not considered an industry-compatible technique. As mentioned in the introduction, here, it was chosen because of its powerful, yet simple materials composition control, but for future work, it will be important to transfer our results to, e.g., a sputter deposition processes. Circular Pt or W (for industry compatibility) top electrodes (TEs) with diameters of 10 to 100 μm were fabricated by a standard ultraviolet (UV) lithography lift-off process and sputtering. For electrical measurements, devices were contacted in a probe station, where in the case of an Nb:STO BE, rather than using an elaborate via process, the bottom contact was established through conductive Ag paint in contact with the Nb:STO. The complete measured structures thus comprised probe tip–TE–thin film–Nb:STO substrate–Ag paint–probe tip, as illustrated in the inset of Fig. 1A. For devices with TiN BEs, a small area of TiN was masked off during film deposition so that the TiN could be contacted directly in the probe station. For all probe station measurements, voltages were applied to the TE with the BE grounded and currents were measured at the TE.

For reference, it is established in the literature that metal contacts deposited directly on Nb:STO can lead to a substantial hysteresis in the current-voltage (*I*-*V*) curves, e.g., due to charge trapping (*25*) or proton exchange (*26*), and in fig. S1, measurements are presented on a Pt-on-Nb:STO reference sample, which, except for thin film deposition, underwent the same fabrication process steps as the devices presented here. It is evident that the shape of the reference *I*-*V* curves is consistently and markedly different from the nanocomposite films discussed in the following. In particular, the endurance and retention performances are much worse for the Pt-Nb:STO devices compared with the nanocomposite films, and higher voltages are required to switch the former.

The best electrical performance was achieved for the nanocomposite devices based on thin films with thicknesses between 15 and 25 nm, deposited at 400°C. (Reference films will be discussed later.) The initial five *I*-*V* curves for each of 10 different pristine devices on the same sample are presented in Fig. 1A and endurance data for 5 of these devices and 5 devices from a second sample are presented in Fig. 1B, where every single switching cycle is recorded as recommended in (*21*). Histograms of the measured endurance performance are provided in fig. S2, and the *I*-*V* curves for the second sample are provided in fig. S3A and had the same shape as in Fig. 1A, only with slightly lower currents. Figure S3B also includes *I*-*V* curves for a sample with W TEs, and their shapes are very similar to the ones in Fig. 1A, too, but the off-state current is higher than in the samples with Pt TEs. A comparison of *I*-*V* curves from devices with Ba:HfO*_x_* films and pure HfO*_x_* films of similar thickness in fig. S4 clearly demonstrates that the *I*-*V* curves based on devices with pure HfO*_x_* films are more insulating, noisy, and not reproducible. Last, fig. S3C presents hysteretic *I*-*V* curves for a sample with a TiN BE instead of Nb:STO. While these *I*-*V* curves are less uniform and more noisy, they prove that the observed RS is not originating from the Nb:STO alone but is a combination of film and electrode. Further optimization of the TiN can be expected to result in the same highly uniform performance as the model Nb:STO electrode. For example, initial experiments suggest that the surface roughness of the TiN has a critical impact on the device reliability, as already during initial iterations, in-house deposited TiN provided better results than commercially available TiN wafers. However, an optimization for complete back end of line compatibility is beyond this initial study of the proposed materials concept.

While it is clear from the *I*-*V* curves in Fig. 1A that the devices undergo a forming step during the first voltage application, this forming does not require a higher voltage than the subsequent switching voltages. (The forming step will be discussed in a later section along with the subsequent RS mechanism.) The switching voltages for all *I*-*V* and endurance measurements were ±2 V. This is lower than the ≈±4 V of other recently reported hafnium-oxide-based multilevel RS devices (*15*, *27*), and approaching the ∼1 to 2 V of state-of-the-art DDR SDRAM (*28*).

The results in Fig. 1 (A and B) demonstrate small cycle-to-cycle and device-to-device variability as well as reliable sample-to-sample reproducibility. Of the 51 devices measured across the two samples from Fig. 1, 75% exhibited stable hysteretic *I*-*V* curves and 71% out of these could maintain a memory window ≈10 for up to 10^4^ switching cycles, as presented in Fig. 1B. In a dedicated cleanroom fabrication environment (unlike a multiuser, multiprocess university laboratory), these numbers can be expected to increase further. For the same reason of a nonideal university fabrication environment, the yield of devices with stable electrical performance decreased for devices with electrode diameters smaller than the 50 and 100 μm in Fig. 1. Nonetheless, similarly uniform and stable endurance performance for devices with 20-μm diameters is presented in fig. S5 with the same switching voltages and memory window as the larger devices in Fig. 1. Furthermore, to explore the extremes of the scalability limits, an area of ca. 100 nm by 100 nm was made conductive (“formed”) inside an otherwise insulating area of the film by conductive atomic force microscopy (CAFM; fig. S6C), and a separate single scan of the film surface reveals that nanometer-sized conductive areas can be formed, only limited by the CAFM tip diameter of 25 nm (fig. S6B).

Because the endurance data were measured with a standard Source/Measure Unit (Keysight B2912A), the widths of the write and read pulses (see inset of Fig. 1C) were on the order of milliseconds. To test the limits of the switching speed, we measured the effect of write pulses with different lengths with a Keithley 4200A-SCS and a Keysight B1500 parameter analyzer and the results are presented in Fig. 1C. For this measurement, the width of the write pulses was changed as displayed on the *x* axis of Fig. 1C, and the width of the subsequent read pulse was kept on the order of milliseconds. A memory window of ≈10 is maintained for write pulse widths down to 100 ns, and a degradation is only observed for write pulse widths of 20. As the measurement setup used DC probe tips and electrode layouts, it is likely that the degradation of the memory window for write pulses of 20 ns is actually caused by the measurement setup and electrode design rather than the switching mechanism itself (*29*). This is further supported by the time-resolved current data during fast measurements, presented in fig. S7, where the current during switching is completely dominated by a typical ringing due to *RC* time constants in the signal path. The separate read pulses with longer rise time and pulse width prove that the devices switch regardless of the ringing and once the ringing subsides at switching voltage rise times of 1 μs, fig. S8, the current follows the voltage instantaneously. For proper fast real-time current-versus-time measurements, dedicated probe pads and tips are required, as demonstrated in, e.g., (*17*), but the development of a corresponding fabrication process was beyond this study.

**Fig. 2.**
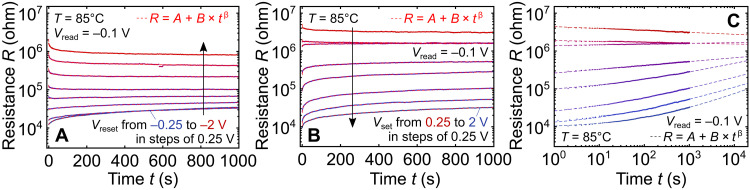
Multiple-state retention measurements at *T*=85°C. (**A**) Negative reset voltages and (**B**) positive set voltages; same device in (A) and (B). In between set/reset operations, the device was reset/set with ∓2 V. In a limited range of the measured 1000 s, the states can be clearly distinguished. Dashed red lines on top of the differently colored measured traces are fits to the power law *R* = *A* + *B* × *t*^β^ with *A*, *B* > 0 and ∣β∣ < 1 (average adjusted *R*^2^ ≥ 0.93). (**C**) Same as (B) but with a logarithmic time axis.

As the final standard figure of merit for RS devices, the state retention at different temperatures is presented in Fig. 2. In Fig. 2 (A and B), at a sample temperature of 85°C, eight different reset and set voltages were applied with negative and positive polarity, respectively, which resulted in seven different states for either polarity, as two states were indistinguishable in either case. For negative reset voltages, the device was set with 2 V in between set operations, and for positive set voltages, it was reset with −2 V in between set operations. In a limited range of the measured 1000s, the different resistance states can be distinguished clearly, and their spacing suggests that additional intermediate states can be programmed. Note that the different resistance states were achieved purely on the basis of voltage application and no current compliance was required, as is often the case for multilevel performance in filamentary devices (*30*).

For nonvolatile memory applications, the typically required (extrapolated) retention time is 10 years. The measured results in Fig. 2 (A and B) can be fitted with a power law of the form *R* = *A* + *B* × *t*^β^ with ∣β∣ < 1. The average adjusted *R*^2^ for these fits is ≥0.93 with *A* and *B* restricted to positive values that can be identified with the measured resistances. This way, the fitted equations can be identified with a physical processes, the Curie–von Schweidler law, which is understood to occur because of time-dependent charge redistribution in dielectrics, e.g., because of charge trapping/detrapping or ionic motion (*31*). Under the assumption that this process governs the state decay, from Fig. 2C, it is clear that at elevated temperatures, the different resistance states cannot be distinguished for the required 10 years. Further temperature-dependent retention measurements in fig. S9 indicate that temperatures as low as 132 K would be required for 10-year data retention.

While this renders the devices unsuitable for conventional data storage, the observed state decay may actually be of advantage for neuromorphic applications (*32*), where especially the rapid initial state decay can be identified with “forgetting” (*33*) or “short-term plasticity” (*34*). Thus, in the following section, we demonstrate neuromorphic functionality of the presented devices, where nonvolatility is not as critical, or sometimes not even as desirable as for data storage applications.

As discussed in the introduction and in, e.g., (*35*), a viable and oft-used way to enhance RS functionality is adding additional layers to the switching stack, and this could be a way to improve the state retention of the devices presented here for conventional memory applications. From the measured retention and the mechanism investigations later on, the state retention seems to be limited by the drift of ions, so it could be improved by an ion-blocking interlayer. As the addition of Ba to HfO_2_ reduces the relative oxygen content of the films (see the “Materials design and properties” section), this interlayer could simply be pure HfO_2_ or alternatively SiO_2_, which has been used before (*35*).

### Neuromorphic functionality

Neuromorphic, or “brain-inspired,” computing is one of the most promising approaches to overcoming the von Neumann bottleneck, i.e., the bandwidth limitation between memory and processor. In a biological brain, information is stored in the strength (or “weight”) of synapses, which connect neurons, and learning (and forgetting) is consequently realized by changing the strength of these connections (*36*). One widely accepted model to achieve such changes is called spike timing–dependent plasticity (STDP) (*33*), which refers to the timing of electric pulses being created in neurons on either side of a synapse. In terms of nonbiological electrical devices such as RS devices, the synaptic strength can be modeled as the controllable resistance of such a device. The following results are based on the more industry-compatible devices with W TEs.

**Fig. 3.**
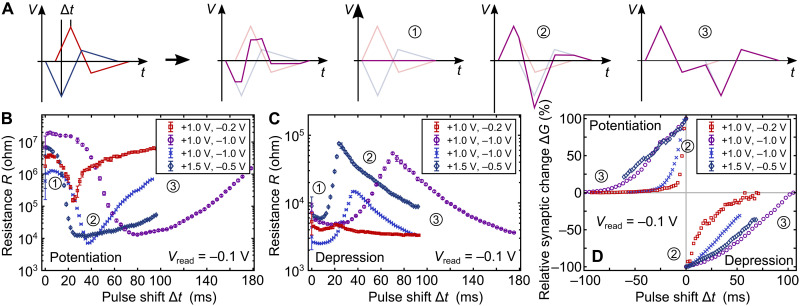
Neuromorphic capabilities. (**A**) Schematic of the neuromorphic voltage profiles: Two mirrored voltage profiles with a total period of ≈150 ms are shifted consecutively as displayed on the *x* axes of (B) and (C). The two values in the figure legends of (B) and (C) correspond to the amplitudes of the two triangular parts of the voltage profiles. The profile with an initial negative amplitude (blue) corresponds to the presynaptic pulse. After shifting, the two profiles are added and applied to the TE. The subfigures marked ① to ③ illustrate the three “extreme” cases where the two voltage profiles add to zero, to the maximum voltage across the device, and two separate profiles, respectively. (**B**) Synaptic potentiation (decrease of resistance) as a result of the applied voltage profiles for three different devices. The difference between the measurements with the same ±1 V voltage amplitudes was the profile width. (**C**) Synaptic depression (increase of resistance) as a result of the applied voltage profiles. From (B) and (C), it is clear that potentiation has a larger dynamic range than depression by a factor of at least 10. (**D**) Relative synaptic weight change normalized to the minimum (maximum) resistance for potentiation (depression) with Δ*t* = *t*_pre_ − *t*_post_ and Δ*t* = 0 corresponding to this minimum (maximum) in (B) and (C), respectively. This figure only takes into account the weight change after the minimum/maximum in (B)/(C) due to the choice of the Δ*t* = 0 point.

Figure 3 (B and C) presents potentiation (reducing resistance → “strengthening connection”) and depression (increasing resistance → “weakening connection”) for four different devices and voltage profiles. The pre- and postsynaptic voltage profiles to emulate neuronal action potentials consisted of two triangular parts to resemble qualitatively the shape of the biological action potentials identified by Hodgkin and Huxley (*37*): a first triangular part for the action potential itself and a second one for the following hyperpolarization (“recovery”). As only two-terminal devices were measured, rather than applying the voltage profiles physically on either side of the devices, the profile corresponding to the BE was inverted and added to the profile of the TE. Before addition, the two profiles were systematically shifted with respect to each other in time by an amount Δ*t*, and the resulting resistance was measured as the average of 100 data points at a constant read voltage *V*_read_ = −0.1 V. The construction of these voltage profiles is illustrated in Fig. 3A, and the respective amplitudes for the profiles are provided in the legends of Fig. 3 (B and C) alongside the measured results. The total profile periods were about 150 ms. This is about an order of magnitude slower than typical time scales in biological processes (*38*) but was limited by the measurement setup rather than the tested devices (see Fig. 1C for device speed). The measurement of each time shift was repeated 10 times, and the data points in Fig. 3 (B and C) correspond to the mean value of these 10 repetitions. The error bars were calculated by SE propagation from the SDs of the 100-point averages while neglecting the small error of the read voltage, and the small resulting errors reveal once again the superior device stability. Figure 3D presents the relative synaptic weight change Δ*G* resulting from Fig. 3 (B and C) in terms of the corresponding conductance *G* = 1/*R*. Here, Δ*t* = *t*_pre_ − *t*_post_; for potentiation (Δ*t* < 0) Δ*G* = (*G* − *G*_min_)/(*G*_max_ − *G*_min_), and for depression (Δ*t* > 0) Δ*G* = (*G* − *G*_max_)/(*G*_max_ − *G*_min_). Δ*t* = 0 in Fig. 3D corresponds to the overlap of the first peak of one of the profiles with the second (inverted) peak of the other, i.e., to the largest total voltage across the device. This normalization was chosen to compare whether the shapes of the potentiation/depression curves could be varied by choosing different voltage profiles. While from these initial measurements a systematic trend is not observable, it is clear that the devices are capable of implementing linear potentiation and depression, which is highly advantageous for synaptic functionality (*32*), and this could be stabilized by applying optimized voltage profiles.

Either way, the general capability of the tested devices to emulate STDP is clear from Fig. 3 (B to D) as the voltage profiles with different Δ*t* result in gradually tuneable resistance states. As expected from the multiple resistance states in Fig. 2, the largest change in resistance is achieved when the first positive peak of one of the voltage profiles coincides with the (inverted) negative peak of the other profile, i.e., the maximum total voltage across a device. This is indicated by ② in the figures. The further away from this condition the two profiles are with respect to each other, the smaller the total voltage across the device and the smaller the change in resistance. This applies both for potentiation and depression. The two “extrema” in these cases are indicated by ① and ③ in the figures, which is when the opposite profiles cancel each other and are completely separated, respectively.

Similar neuromorphic functionality based on hafnium oxide thin films has only been demonstrated in devices with additional layers in the oxide stack, both for gradual (*12*, *13*, *15*, *27*) and filamentary (*30*) switching, or in ferroelectric systems (*39*), all of which require more stringent materials control than the devices presented here. The demonstration of STDP presented here opens up a whole range of more intricate learning implementations for future work and demonstrates an easy-to-fabricate synapse device based on amorphous hafnium oxide.

### Materials design and properties

To elucidate the relation between the electrical performance and the materials properties, we fabricated reference devices based on different thin film deposition conditions. With these references, the separate effects of Ba addition and deposition temperature on the film crystallinity were studied.

Main system of study: Ba:HfO*_x_* deposited at 400°C (PLD target Ba:Hf 1:2).

Reference samples:

(i) Pure HfO*_x_* deposited at 400°C.

(ii) Pure HfO*_x_* deposited at 30°C.

(iii) Pure HfO*_x_* deposited at 800°C.

(iv) Ba:HfO*_x_* deposited at 800°C from the same target as the main system of study.

For all reference samples, the *I*-*V* curves were less uniform and less repeatable (see, e.g., fig. S4) than for the Ba:HfO*_x_* films deposited at 400°C (main system of study), and only a small percentage of measured devices maintained a memory window of ≥10 for ≥10^3^ switching cycles. In light of this instability at the level of *I*-*V* curves and endurance, retention was not characterized. The only outlier in this regard was the Ba:HfO*_x_* film deposited at 800°C, which maintained a much more stable retention (see fig. S10, memory window >10^4^ for >10^5^ s at room temperature) than devices based on any of the other films, but the high deposition temperature renders it unsuitable for CMOS integration.

**Fig. 4.**
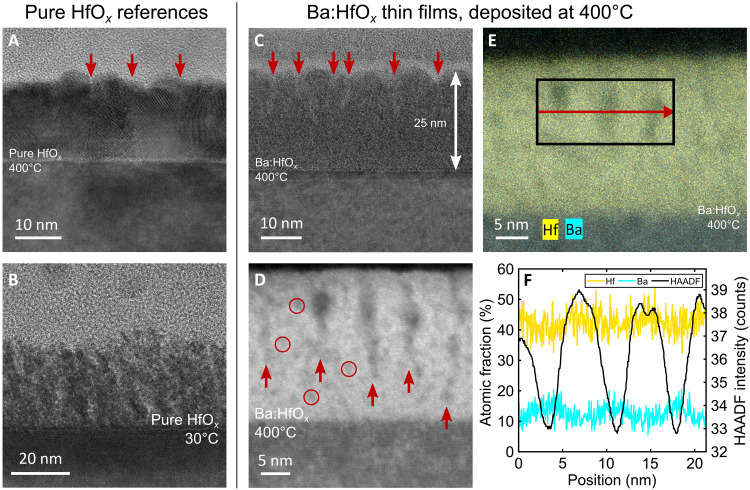
Cross-sectional TEM images and energy-dispersive x-ray measurements from high-angle annular dark-field scanning TEM for different thin films. (**A**) Pure HfO*_x_* deposited at 400°C. Clear crystallites are visible in the film; red arrows indicate some of the grain boundaries. (**B**) Pure HfO*_x_* deposited at 30°C. While these films are not polycrystalline like pure HfO*_x_* deposited at 400°C, neither are they as uniform as the composite films presented in (C). (**C**) The thin films which resulted in stable electrical performance are amorphous or nanocrystalline. Some pillar-like structures can be discerned, indicated by red arrows. The addition of Ba to the films clearly leads to material uniformity by suppressing crystallization. (**D**) High-angle annular dark-field scanning TEM (HAADF-STEM), zoomed in on some of the pillars. In addition, darker nanoparticles can be discerned throughout the films; four randomly chosen particles are marked by red circles. (**E**) HAADF-STEM image to indicate the area scanned for EDX and the elemental distribution of Hf and Ba. (**F**) Line scan EDX results acquired from the area indicated in (E). The dark areas in the HAADF-STEM image contain more Ba than the brighter ones. The ratio between Ba and Hf in the pillars is about 0.25 to 0.33, consistent with the Rutherford backscattering analysis discussed later.

The main effect of the Ba addition to hafnium oxide can be discerned from the cross-sectional transmission electron microscopy (TEM) images in Fig. 4, which compares the microstructure of the different films. The reference films of (i) pure HfO*_x_* deposited at 400°C (Fig. 4A) and (ii) pure HfO*_x_* deposited at 30°C (Fig. 4B) are structurally much less uniform than the Ba:HfO*_x_* film, as is evident when comparing them with Fig. 4C. For reference (i), large crystallites with different lattice orientations are visible with grain boundaries reaching throughout the whole thickness of the film. For reference (ii), the cross-sectional TEM shows an amorphous, but very rough and irregular texture.

In the Ba:HfO*_x_* film deposited at 400°C (Fig. 4C), such features are completely absent and the film appears amorphous, very smooth, and regular. However, there are vertically aligned regions with a pitch of ≈5 to 10 nm and widths of ∼2 nm embedded in the regular host matrix and penetrating at least two-thirds of the way through the film. These columns are indicated by red arrows in Fig. 4 (C and D), and to distinguish them more clearly, Fig. 4D presents a high-angle annular dark-field (HAADF) scan of the film, where the vertical nanocolumns appear darker than the surrounding matrix. Besides the nanocolumns, it also reveals the presence of similar darker regions in other parts of the film as ∼2-nm-wide nanoparticles, some of which are indicated by red circles in Fig. 4D. Neither the host matrix of the films nor the darker regions are crystalline, which explains why neither phase can be observed by x-ray diffraction (XRD) measurements (fig. S11C). The darker color in the HAADF scans indicates a higher concentration of a relatively lighter element and the energy-dispersive x-ray (EDX) diffraction measurement presented in Fig. 4 (E and F) confirms that these regions are Ba-rich compared with the surrounding matrix. This local Ba segregation as well as the overall film amorphization can be explained by the Ba solubility in crystalline HfO*_x_* being exceeded, which destabilizes any crystalline phase and leads to the forming of a separate amorphous Ba-rich phase (*22*). The limited Ba solubility in HfO*_x_*, together with a reported Ba volatility especially during low-temperature PLD (*40*, *41*), can explain why the observed nanocolumns only start forming after a certain film thickness has been reached. During deposition of the first few layers, the low Ba solubility in HfO*_x_* favors a Ba segregation to the top of the films, and some of it may be lost to the deposition atmosphere. Only after a certain amount of Ba has been incorporated into the films and sufficient regions of the second Ba-rich phase have formed can they coalesce into the observed nanocolumns.

From Fig. 4F, the Ba cation fraction in the matrix is about 10/(10 + 45) ≈ 0.18 and it is higher in the nanocolumnar second phase region, varying between ≈0.25 and ≈0.33. Because the nanocolumnar regions are only a few nanometers in diameter, EDX scans across them will also sample some of the matrix in which they are embedded. Thus, the local Ba content in the nanocolumns cannot be measured to a high degree of accuracy and is likely higher than what can be concluded from Fig. 4F. Irrespective of the exact materials composition, the higher Ba content in the amorphous columnar regions is evident.

The fine nature of the second phase interspersed in the Ba:HfO*_x_* matrix is expected at the deposition temperature of 400°C, where the diffusion kinetics are insufficient for long-range atomic diffusion or the formation of crystalline material. The columnar regions are formed where short-range diffusion kinetics are sufficient for nanoparticle regions to coalesce. As will be discussed in a later section, these second-phase columnar regions can facilitate RS and electronic conduction.

The strong suppression of “large” (on the order of the film thickness) crystallite formation is evident also at the high deposition temperature of 800°C, which is documented in fig. S11 (XRD) and S12 (TEM). Again, pure HfO*_x_* forms clearly polycrystalline films, whereas films deposited from the composite target form tiny (order of unit cell) nanocrystals at best or most likely are amorphous. From these TEM studies, it can be concluded that the addition of Ba has a stronger effect on the materials amorphization than the deposition temperature.

**Fig. 5.**
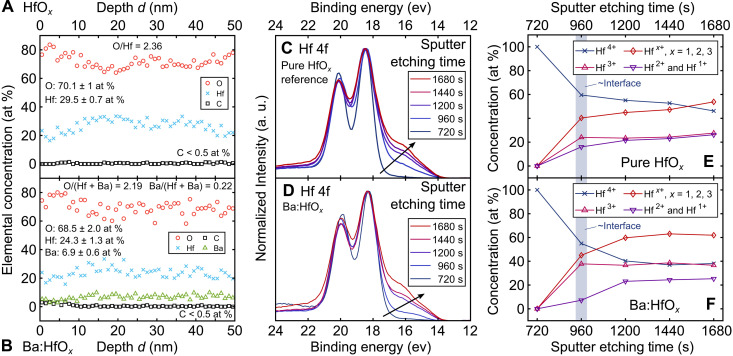
ToF-ERDA and depth-resolved XPS. (**A** and **B**) ToF-ERDA of the HfO*_x_* references (A) and Ba:HfO*_x_* films (B). The relative oxygen content in the Ba:HfO*_x_* films is lower than in the pure references, and the only detected impurity trace is less than <0.5 at.% C in either film. (**C** and **D**) Hf 4f core spectra for the HfO*_x_* and Ba:HfO*_x_* sample, respectively, from which the relative concentrations of different oxidation states in (E) and (F) were derived. Corresponding spectra for Ba 3d and O 1s can be found in fig. S14. The increase in Hf suboxides with increasing etching time can be derived from the increasing low-energy shoulder. a. u., arbitrary units. (**E** and **F**) Relative concentrations of hafnium oxidation states for (E) a pure HfO*_x_* reference and (F) a Ba:HfO*_x_* film. The *x* axes start at 720 s as this was the time required to etch a protective TiN top layer. This means that the actual film surface is reached after about *t* = 960 s (indicated by blue shading), and in this area, different Hf-N-O compounds were detected. At *t* = 1680 s, the etching approached the substrate. The most notable difference between the two samples is the amount of Hf suboxidation states, i.e., Hf^*x*+^ with *x* < 4, in the composite films with a maximum of about 40 to 55% in pure HfO*_x_* (E) versus a maximum of up to 63% relative concentration in Ba:HfO*_x_* (F).

For a detailed analysis of the materials compositions and especially the effect of the added Ba, time-of-flight elastic recoil detection analysis (ToF-ERDA) and Rutherford backscattering spectrometry (RBS) were carried out on Ba:HfO*_x_* nanocomposite films and pure HfO*_x_* reference films, all deposited at 400°C on commercial 200-μm-thick thermally formed SiO_2_ on top of a Si substrate; for either material, films were deposited with ≈15-nm thickness, similar to the electrically measured devices, and with ≈120/100-nm thickness to evaluate the homogeneous elemental distributions and the effect of electronic stopping cross sections on the film compositions (*42*). In addition, the chemical bonding and oxidation states of Ba:HfO*_x_* and HfO*_x_* thin films on top of Nb:STO were analyzed by depth-resolved x-ray photoelectron spectroscopy (XPS) and compared with reference films deposited on commercial Si. The ToF-ERDA and XPS results are summarized in Fig. 5 with further XPS details provided in fig. S14, and the RBS results are presented in fig. S13 and table S1.

On the basis of RBS, the thin and thick films of pure HfO*_x_* yielded compositions of HfO_2.39_ and HfO_2.41_, respectively, and for the Ba:HfO*_x_* films the compositions were Ba_0.21_Hf_0.79_O_2.15_ and Ba_0.21_Hf_0.79_O_2.17_ for the thin and thick film, respectively. On the basis of ToF-ERDA, the film compositions (for the thick films) were HfO_2.36_ and Ba_0.22_Hf_0.78_O_2.19_. The close agreement in compositions obtained from films with different thicknesses and from different experimental techniques proves a highly accurate quantification and homogeneous elemental distributions throughout the films with no effects of electronic stopping cross sections on the film compositions. Furthermore, the Ba fractions in Ba:HfO*_x_* as obtained from the two techniques is close to that measured by EDX; cf. Fig. 4F. The Ba deficiency with respect to the PLD target composition of Ba:Hf 1:2 can be explained by the volatility of Ba during low-pressure deposition, especially at temperatures below 600°C (*40*, *41*).

On the basis of the total Ba/Hf fraction of ≈27% in the films (from RBS, table S1) and the HfO*_x_* host matrix making up ≈70 to 80% of the total film volume fraction *V*_f_ (from Fig. 4D) with a Ba/Hf fraction of 10/45 ≈ 0.22 (from EDX, Fig. 4F), the Ba/Hf cation ratio α in the nanocolumns can be estimated from α*V*_f_(columns) + 0.22*V*_f_(matrix) = 0.27, which results in α ≈ 0.4 to 0.5. This implies that the nanocolumns most likely consist of amorphous BaHfO_3_ (rather than BaO).

As to the oxygen composition of the films provided above, RBS and ToF-ERDA consistently revealed an excess of oxygen compared with stoichiometric films for the four different films studied (HfO*_x_* and Ba:HfO*_x_* films of two different thicknesses each, Fig. 5 and fig. S13), and such excess oxygen has been reported in hafnium oxide films both experimentally (*43*) and theoretically by density functional theory (*44*). As neither RBS nor ToF-ERDA detected any significant amounts of impurities that could explain the excess oxygen, the most likely cause here is the presence of cation vacancies. These cation vacancies can be expected to form both in amorphous hafnium oxide, especially under oxygen-rich deposition conditions (*45*), and in the Ba-rich second phase, which is most likely BaHfO_3_ (*46*, *47*). We note that the presence of oxygen interstitials in the film could provide an additional explanation for the measured excess oxygen (*45*), but the presence of cation vacancies is a more conventional explanation. Irrespective of the exact reason for the excess oxygen, the key point about the oxygen content in this work is its relative change upon the addition of Ba (≈O_2.39_ for HfO*_x_* versus ≈O_2.17_ for Ba:HfO*_x_*), which was measured consistently by both RBS and ToF-ERDA. For both the thin and the thick films studied, the oxygen content is about 10% lower in the films containing Ba. This is expected, because if a lower-valent ion substitutes for a higher-valent ion (i.e., Ba^2+^ instead of Hf^4+^), a loss of oxygen is necessary for charge compensation.

To conclude the compositional analysis, depth-resolved XPS was carried out to reveal whether the addition of Ba to hafnium oxide influences the chemistry or oxidation states of the different elements in addition to suppressing crystallization. Details of how the XPS data were fitted are provided in fig. S15. The major difference between HfO*_x_* and Ba:HfO*_x_* in this analysis is the increase in the relative concentration of Hf suboxidation states, i.e., Hf^*x*+^ with *x* < 4, from 40% in pure HfO*_x_* to about 63% in Ba:HfO*_x_*. Because the HfO*_x_* conduction band is predominantly formed by the Hf 5d orbitals (*48*), the higher electron density resulting from the increased concentration of Hf suboxidation states may well contribute to the higher conductivity of the Ba:HfO*_x_* films, which was pointed out earlier.

For the XPS experiments, the Ba:HfO*_x_* and reference HfO*_x_* thin films were deposited in the same way as the films for the RS devices, but instead of depositing TEs, they were capped in situ (i.e., without venting the PLD chamber) with a TiN layer to avoid any initial core level change (“damage”) induced by Ar ion sputter etching during the XPS measurements. Figure 5 (C and D) presents the Hf 4f core level spectra obtained from the reference HfO*_x_* and Ba:HfO*_x_* films, respectively, as a function of sputter-etching time *t*, starting at *t* = 720 s, which corresponds to the time required to remove most of the protective TiN layer. At this point, the top surface of the oxide thin films could be measured before the onset of sputter damage that changes the chemical bonds. The actual surface of the oxide thin films is thus reached after about *t* = 960 s as indicated by the shaded area in Fig. 5 (E and F). Ba 3d and O 1s spectra corresponding to the Hf 4f ones are provided in fig. S14. The peak intensities of all spectra are normalized, and the energy shifts in the peak positions were corrected to compare changes in the shapes of the spectra.

The Hf 4f spectra acquired from both samples (HfO*_x_* and Ba:HfO*_x_*) at *t* = 720 s consist of 4f7/2 and 4f5/2 peaks, which are fitted well with a single doublet pair corresponding to fully oxidized Hf, i.e., Hf^4+^. The broader spectra obtained for *t* ≥ 960 s, however, can only be fitted as convoluted spin-split 4f7/2-4f5/2 doublet electron states, and the doublets at lower binding energies reveal the additional presence of Hf^3+^.

In addition to the peak convolution, a large shoulder with lower intensity starts appearing at lower binding energies for the Hf 4f spectra of both films, which reveals the presence of Hf^2+^ and Hf^1+^ oxidation states. For increased sputter times, in addition to a gradual peak broadening of the 4f7/2-4f5/2 doublets, the area below this shoulder increases. Both observations can be attributed to sputter damage induced by the Ar ion etching (*49*), but as evidenced by further reference measurements discussed in more detail with fig. S14, this is not the only cause in the samples investigated here. For the reference measurements on a thick film of pure HfO*_x_*, for increasing etching times, the shape and area of this low energy should remained constant. Thus, the gradual increase of the shoulder area as a function of depth observed in Fig. 5 reveals a changing concentration of Hf suboxides intrinsic to the films deposited on Nb:STO.

This change is resolved in Fig. 5 (E and F) as a function of the sputter etching time *t* and thus film depth for the reference HfO*_x_* and Ba:HfO*_x_*, respectively. In general, for both materials, the Hf^4+^ concentration decreases with increasing depth, while the total concentration of Hf^*x*+^ with *x* = 3,2,1 increases. While the two figures look very similar because of the normalization, critically, a detailed comparison of how the Hf^*x*+^ concentration changes in the two materials reveals that the addition of Ba to HfO*_x_* increases the relative amount of sub oxides Hf^*x*+^ with *x* < 4 from about 40% in pure HfO*_x_* to about 63% in the Ba:HfO*_x_* nanocomposite. As mentioned before, this is expected as the lower-valent Ba^2+^ substituting on the Hf^4+^ site leads to oxygen loss to achieve charge compensation and the resulting higher electron density can lead to increased film conductivity. Oxygen vacancies and other materials defects are well known to act as charge traps (*50*) and to affect electronic conductivity in hafnium oxide (*51*). This will be important later on to explain the observed stable RS.

Similar changes as for the Hf spectra would be expected in the Ba 3d and O 1s spectra. However, after normalizing the peak intensities and correcting the peak position shifts, all Ba 3d and O 1s spectra of both oxide films maintain very similar peak shapes with no detectable peak splitting (fig. S14). This can be attributed mainly to the peak positions of Ba 3d and O 1s with different oxidation states being very close, and the changes in the concentration of their oxidation states not being sufficiently high in the films to result in a visible variation in peak shapes.

### Proposed model for the switching and conduction mechanisms

In the following, on the basis of a number of key experimental observations, we will provide a model for the switching and conduction mechanisms that can explain consistently all of these observations. First of all, it can be concluded that the switching mechanism is not filamentary. This is because: (i) The switching currents at +2 V and the corresponding resistances increase/decrease linearly with the electrode diameter (fig. S16). This excludes RS due to a single filament, as in this case, there would be no scaling with the electrode diameter. At the same time, the full device area does not seem to be involved, as in this case, the current should scale linearly with the electrode area instead of the diameter (*52*). In anticipation of the explanation following below, the most likely reason for this “in-between” observation is the growth of Ba-rich nanocolumns with different lengths, which can be seen in Fig. 4D. When the nanocolumns establish preferred electronic conduction paths during the forming step, columns of different lengths contribute differently to the total current, and a certain distribution of column lengths can explain the apparent diameter dependence. (ii) Neither during forming nor subsequent switching is the typical ultra-nonlinear current increase observed, which is characteristic of filamentary switching. (iii) For complete filaments reaching through the whole film, the device resistance should be dominated by the BE series resistance. On the basis of the resistivity of the Nb:STO BE and the device sizes, the measured resistances (fig. S16B) are more than an order of magnitude too high to originate from the BE resistance. For the measured electrode diameters of 25 to 100 μm, the Nb:STO should only contribute a maximum of 60 to 3 ohm to the total resistance, whereas the measured resistances are several hundred ohms up to >1 kilohm. The resistance of the Ag paint was measured as a few ohms.

Instead of being determined by a filament, the resistance states are controlled by a Schottky-like energy barrier. This is evident from an analysis of the electronic conduction, i.e., the measured *I*-*V* curves at different temperatures from 125 to 360 K (≈85°C, fig. S17). With details provided in the Supplementary Materials (“Fitting of electrical data”), they can be fitted very consistently with different thermionic emission models. The best fits, i.e., over the largest current ranges both for positive and negative bias in all RS devices (>50 across three samples) are consistently achieved with the Schottky emission model for a reverse-biased Schottky contact (*53*) at low to medium currents, and with a trap-assisted tunneling (TAT) model (*54*) or a space charge–limited conduction (SCLC) model in the presence of charge traps (*55*) for the highest currents. It is pointed out explicitly that the expression for a forward-biased Schottky contact [∝ exp (*V*) instead of $\propto \;\exp(\sqrt{V})]$ does not fit the measured *I*-*V* curves.

Because several thermionic current mechanisms have a similar exponential dependence on the applied electric field, the good fits by the Schottky emission model are not a claim to the singular presence of this mechanism. At different current levels, other thermionic mechanisms such as Poole-Frenkel (PF) emission or TAT can describe intervals of the *I*-*V* curves as well, and it is very likely that different mechanisms are at play at the same time. Similar arguments hold for the TAT/SCLC fits at the highest current levels.

For the sake of argument, the Schottky emission model can be used to illustrate how the changing energy barrier height sets the resistance states. For the 10 devices in Fig. 1A, the calculated Schottky barrier heights are 0.65 ± 0.03 eV and 0.62 ± 0.03 eV for the high resistance state (HRS) in the positive and negative voltage directions, respectively, and 0.46 ± 0.03 eV and 0.47 ± 0.03 eV for the low resistance state (LRS) in the positive and negative voltage ranges, respectively. The other >40 devices across different samples yielded virtually the same values, and while with different absolute values, PF emission and TAT yielded qualitatively similar changes for the respective barrier heights.

In the double-logarithmic plot with absolute current values in Fig. 1A, the switching polarity is counterclockwise for both positive and negative voltages (counterclockwise/clockwise if the current is not in absolute values). If charged particles such as oxygen vacancies or electrons are involved in the switching process, which is widely assumed for bipolar RS and further supported by the observed state decay following the Curie–von Schweidler law, then this allows only two scenarios. If only one type of charged particle is involved, then only one barrier can dominate the resistance. If the resistance is controlled by two barriers, then different charged particles with opposite sign have to be involved. With the almost completely symmetrical values for the barrier heights under forward and reverse bias calculated from the thermionic emission models above, this means that either there are two barriers of the same height or there is only one effective dominant barrier.

By varying the materials of the TE and BE, it can be concluded that the switching is controlled by one Schottky-like barrier between the Ba:HfO*_x_* films and the BE. This is because TEs with very different work functions Φ [Pt, Φ_Pt_ ≈ 5.8 eV; W, Φ_W_ ≈ 4.7 eV (*56*)] resulted in very similar *I*-*V* curves, while changing the BE from Nb:STO to TiN requires great care in the optimization of TiN (fig. S3). The reason for the restriction of the RS process to the BE is the increased conductivity of the Ba-rich nanocolumns after the forming step and will be discussed in a following paragraph together with the forming process. As the heavily n-type Nb:STO BE determines that electronic conduction occurs because of electrons (as opposed to holes), the barrier controlling the RS has to be due to the conduction band alignment of the devices, and as discussed immediately above, the resistance control in bipolar switching devices by only one energy barrier necessitates that particles with only one charge polarity are involved in the switching process.

**Fig. 6.**
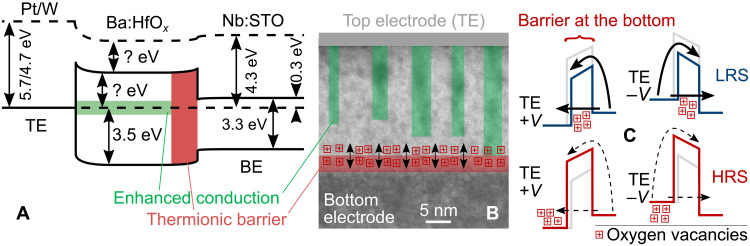
Illustration of switching and conduction mechanisms. (**A**) Schematic band diagram of the RS devices. The values for the metal work functions are taken from the literature. The difference between the Fermi level and the Ba:HfO*_x_* conduction band as well as the Ba:HfO*_x_* electron affinity are marked as unknown, as they could not be measured and the uncertainty of their values does not allow estimating a nominal barrier height for electronic conduction. All other provided values were measured. (**B**) Cross-sectional TEM, same as Fig. 4D, but overlaid with colors to indicate the spatial locations of the different switching components. Green, Ba-rich enhanced conduction channels. Red, thermionic barrier that controls the resistance states. Squares with plus signs, oxygen vacancies move toward and away from the barrier and thus change its height. (**C**) Schematic of electronic conduction in the LRS and high-resistance state (HRS). The voltage-driven accumulation of oxygen vacancies in or close to the barrier area decreases (blue, top) the barrier height, the depletion of oxygen vacancies increases it (red, bottom). Gray references for comparison with the respective other barrier shape. With the partial filament defect bands [green in (A) and (B)] “short-circuiting” the TE/Ba:HfO*_x_* barrier, the barrier area shaded in red in (A) and (B) acts as the limiting barrier for both positive and negative voltage polarity, and it appears almost symmetric from either direction because of the position of the defect band. (There is still a difference of a factor ≈10 in the maximum currents in positive and negative voltages.)

For an accurate illustration of the energy barrier controlling the RS, the band alignment of the devices was measured by UV photoelectron spectroscopy (UPS; fig. S21) and UV-visible spectroscopy (UV-vis), and the results are presented in Fig. 6A. UV-vis with wavelengths between 280 and 1300 nm was used to measure the Nb:STO bandgap (≈3.3 eV); the Ba:HfO*_x_* bandgap is larger than 4.43 eV, which was the maximum measurable value for the used wavelengths.

Last, again on the basis of the switching sequence of the *I*-*V* curves (switch to LRS at positive voltages, switch to HRS at negative voltages), the observed RS cannot be due to electron trapping. This is because a positive voltage at the TE would lead to electron trapping from the Nb:STO into the Ba:HfO*_x_*, which would increase the energy barrier for electronic conduction, thus leading to the HRS instead of the LRS. Conversely, a negative voltage at the TE would lead to electron detrapping from the Ba:HfO*_x_* into Nb:STO, thus lowering the barrier height and leading to the LRS instead of the observed HRS.

With charge trapping excluded as the switching mechanism, the next-likely species to be involved in the switching are oxygen ions or vacancies and with oxygen vacancies being the more commonly evoked species to explain RS in the literature; a summary of the derived RS process is presented in Fig. 6 with oxygen vacancies illustrating the charges that cause switching. Note, however, that it may just as well be oxygen ions that move during switching and the choice for Fig. 6 is mainly illustrative. Oxygen (vacancies) being the moving species is further supported by the observation that Ti TEs result in purely resistive *I*-*V* curves without any RS (fig. S3). As Ti is a strong oxygen scavenger, this confirms that the concentration of oxygen (vacancies) is important for the RS.

In summary, the RS in the presented devices is controlled by a conduction band energy barrier between the thin films and the BE. A positive bias at the TE drives oxygen vacancies into this barrier region (draws oxygen ions out), which lowers the energy barrier (*57*) and leads to the LRS. Conversely, a negative bias at the TE draws oxygen vacancies out of the barrier region (drives oxygen ions into the region), which increases the barrier height and leads to the HRS. Intermediate resistances states (Fig. 2) can be explained by different amounts of vacancies/ions in the barrier region, and the power-law state degradation can be explained by a concentration gradient–induced drift of vacancies/ions after the switching voltage is removed.

Before explaining the forming process as the final piece of the mechanism, the seeming discrepancy between ionic/vacancy movement and the observed low-temperature RS (fig. S17) can be reconciled by results from the literature. While oxygen (vacancy) movement in (amorphous) hafnium oxide decreases rapidly with decreasing temperature (*58*), it has been shown that the presence of an electric field vastly reduces the required activation energy for such movement and also for the generation of additional vacancies (*59*). In addition, it is established that locally high temperatures due to Joule heating strongly affect RS and can easily dominate over the ambient temperature (*60*).

Evidently, an irreversible structural change occurs in the devices during the initial low-voltage forming step. In the literature, such changes have been revealed, e.g., as charge trapping–assisted generation of oxygen vacancies (*59*), oxygen loss to the surroundings (*61*), or the generation of other additional defects in the vicinity of preexisting local disorder (*62*). All of these explanations seem feasible for our devices, but further measurements would be required to draw a definitive conclusion.

Irrespective of the exact atomistic mechanism, with the nanoscale CAFM resolution of the forming process in fig. S6 (B and C) and previous reports that preexisting local disorder facilitates the formation of filaments (*62*), it can be concluded that the columnar Ba-rich regions highlighted in Figs. 4D and 6B fulfill their design purpose of acting as such primary locations to guide the controlled formation of preferred electronic conduction channels. While the CAFM tip diameter of 25 nm was too large to resolve individual such nanocolumns, the conductance contrasts within the conductive areas of fig. S6B may well be traces of these individual columns. Note, however, that these nanocolumns do not reach all the way to the BE (Fig. 4D). This spatial limitation of the nanocolumns and the increased film conductivity due to the increased concentration of Hf suboxidation states (Fig. 5) prevent a complete dielectric breakdown as in filamentary devices, and the remaining thin region at the bottom of the thin films that is not penetrated by the Ba-rich nanocolumns is where the energy barrier is formed which controls the RS as explained above. For this reason, we refer to these conduction paths as “partial filaments,” and they fulfill two roles. Their uniform spatial distribution on a nanoscale leads to the uniform electrical device behavior, and they short-circuit any Schottky-like contact between the TE and the Ba:HfO*_x_* to restrict the RS to the bottom interface. As the TEM analysis in Fig. 4 indicates that the initial Ba-rich nanocolumns form with a certain length distribution, this can also explain the switching current dependence on the electrode diameter rather than the area. Areas below the nanoclumns that penetrate deeper into the films will contribute more to the total device current than shorter columns, and similarly, these areas may experience stronger electric fields and thus be modulated more strongly during resistance chances.

The role of the partial filaments as the effective TEs is indicated as a green defect band in Fig. 6 (A and B). The exact position of this defect band in energy was not measured, but it is well established that in hafnium oxide, a wide defect band tends to form slightly above the middle of the bandgap (*63*) and its exact position determines the barrier height for currents under negative voltages at the TE, i.e., the energy offset between the energy level of the filament and the bottom energy barrier.

As a final verification, a rough estimation of the ionic defect concentration involved in changing the barrier height can be calculated. Additional charge at a conduction band Schottky barrier changes the built-in potential *V*_bi_ and thus the barrier height (and width) so that the new built-in potential *V*_bi_′ is (*64*)

$${V_{\mathrm{bi}}}^{\prime }=V_{\mathrm{bi}}-\frac{\sigma }{\varepsilon_{0}\varepsilon_{r}}\delta$$ (1)

where σ is the additional surface charge projected to a distance δ from the interface and ε_0_ and ε_r_ are the vacuum and oxide relative dielectric permittivity, respectively. On the basis of Eq. 1, the effective addition of a positive charge σ near the interface will decrease the barrier height, whereas a negative charge density will increase it. As a plausibility check, with Eq. 1, a value for σ can be calculated that corresponds to the measured difference in the Schottky barrier height between HRS and LRS Δ*V*_bi_ ≈ 0.18 eV = σδ/(ε_0_ε_r_). When assuming δ = 1 nm and ε_r_ = 15 as an estimation for a low-quality hafnium oxide (*65*), σ = 0.024 C/m^2^ = 2.4 μC/cm^2^, which corresponds to a sheet charge density of 1.5 × 10^13^ cm^−2^. This value is well within commonly observed ranges for electrically active defect densities at or close to interfaces between hafnium oxide and a semiconductor (*66*), so this simplified assumption of a changing Schottky barrier height due to the movement of oxygen ions seems physically reasonable. In addition, the value of 2.4 μC/cm^2^ is not far off the polarization values observed during FE switching in hafnium oxide (*20*), which makes sense considering that both in the present amorphous case and in the case of (poly-)crystalline FE switching, the change comprises a certain number of atoms changing their position by just a small distance.

In summary, stable and uniform interfacial RS was engineered in amorphous hafnium oxide nanocomposite thin films and the underlying mechanisms were analyzed in detail. The thin films were deposited at the CMOS-compatible temperature of 400°C and lead to low cycle-to-cycle, device-to-device, and sample-to-sample variability. This was achieved on the basis of the addition of Ba to the system (i) enabling smooth amorphous films; (ii) reducing the oxygen concentration and Hf oxidation states, thus increasing the film conductivity to avoid filamentary dielectric breakdown; and (iii) creating nanocolumns of a Ba-rich second phase that facilitate the formation of only partial filaments and restrict the switching to the bottom interface by “short-circuiting” any top interface Schottky-like barrier. These nano-engineered materials properties are essential for the RS mechanism, which was identified as the adjustable height of an interfacial thermionic barrier by ionic migration. Switching endurances of >10^4^ cycles with a memory window ≥10 and switching speeds down to ∼20 ns were demonstrated for a large number of devices. With the gradual changeability of resistance states, device-inherent STDP was demonstrated as a promising basis for neuromorphic applications. Last, this conceptual demonstration of amorphous phase-separated nanocomposite thin films has the potential to open up a whole range of thin film properties design parameters.

## MATERIALS AND METHODS

### PLD targets

For the pure hafnium oxide target, HfO_2_ powder of purity >99.9% was ground for 40 min, pressed into a pellet, and then sintered at 1400°C for 8 hours. For the composite target, BaCO_3_ and HfO_2_ powders of purity >99.9% were weighed in a stoichiometric ratio, ground for 40 min, distributed on a flat surface, and calcinated at 850 to 950°C for 2 hours. Afterwards, the calcinated powder was mixed with HfO_2_ powder in a ratio 1:1, ground again for 40 min, pressed into a pellet, and sintered at 1250°C for 8 hours.

### PLD of oxide thin films

Except for the temperature, which was varied as described in the main text, the following parameters were used for all depositions. Fluence *F* = 2 J/cm^2^, growth time *t* = 12 min for all samples except for the thicker samples, where *t* = 120 min, frequency *f* = 2 Hz, oxygen pressure *p* = 0.13 mbar, oxygen flow *Q* = 6 sccm (gas at room temperature). After deposition, the heater was cooled down to 200°C at 5 or 10°C/min in the same oxygen atmosphere as during deposition, but without continuous flow, and at 200°C, the heating current was switched off to let the heater cool down on its own.

### Film thickness measurements

To measure the thickness of layers deposited by PLD, some samples had one corner covered with TiO_2_ during deposition. After the deposition, the TiO_2_ was removed and the resulting step height measured by standard tapping mode atomic force microscopy. For all TEM samples, the thickness was also confirmed by TEM.

### XRD measurements

XRD measurements were carried out using a Panalytical Empyrean system with parallel beam optics, CuK_α1_ radiation, a single-point proportional detector, or a PIXcel3D position-sensitive detector.

### Sample fabrication

Two different substrates were used, either Nb:STO or MgO. For Nb:STO, the oxide thin films were deposited directly onto the substrate. For MgO, ≈36 nm of TiN was deposited by PLD (*T* = 650°C, *F* = 1 J/cm^2^, *t* = 50 min, vacuum *p* ≈ 1 × 10^−5^ mbar) as the BE and the films were deposited on top. For all samples, TEs were deposited in a standard lift-off process. The positive UV photoresist AZ 4533 was spin-coated (8000 rpm for 30 s, acceleration time 10 s, deceleration time 5 s) onto the samples surface, soft-baked at ≈100°C for 2 min, and exposed to UV light for 11.5 s through a mask. The resist was developed with the AZ 351 B developer, and the top metal Pt or W was sputtered on. Afterwards, the resist lift-off was carried out by submerging the samples in ethanol for about 20 min and carefully sonicating the containing beaker to remove the unexposed UV resist and lift off the metal on top.

### Electrical measurements

All electrical measurements except for the pulse width dependence were carried out with a computer-controlled Keysight B2912A, connected to an Everbeing manual probe station with triax cables. For the fast pulse-width-dependent measurements, a Keithley 4200-SCS and a Keysight B1500 parameter analyzer with a dedicated ultrafast pulse measure units were used, connected to a manual probe station. For Nb:STO substrates, samples were glued to a glass slide with conductive Ag paste and the Ag was contacted by a probe tip. It was verified that the conductivity of the Ag was only a few ohms.

### Conductive atomic force microscopy

CAFM was carried out on bare areas of a film with W electrodes, i.e., the same film that was used for conventional *I*-*V* measurements. The setup consisted of a Bruker Dimension Icon Pro using a PtIr-coated Sb-doped Si SCM PIC tip in contact mode. The bottom of the sample was electrically connected to the AFM tool by gluing it to a metal disc with conductive Ag paint. Voltages were applied to the bottom of the sample while the CAFM tip was grounded.

### TEM and EDX spectroscopy

Cross-sectional TEM samples were manually fabricated in three steps. First, for each TEM sample, two rectangular pieces (≈1.5 mm × 2.5 mm) cut from the samples of interest were pasted film-to-film with m-bond at 220°C for 4 hours and then cooled to room temperature. The glued sample was then ground with 600 grits, followed by a fine polishing process using silica diamond papers in the sequence of 15, 6, 3, and 1 μm to ensure the flatness of the lamella. Last, the lamella underwent an ion-milling process using a PIPS II precision ion polishing system to reach the thickness required for TEM (≈100 nm). TEM, scanning TEM (STEM), and EDX were performed using a Talos F200X G2 TEM with a gun brightness of 200 kV. Bright-field mode was used to capture TEM images, and HAADF imaging was used for STEM.

### Rutherford backscattering spectrometry

Dedicated samples for RBS were fabricated by depositing films of different thickness (15 and 100/120 nm) on commercial thermally formed SiO_2_ on top of Si. RBS was carried out in a 5-MV 15SDH-2 tandem accelerator to obtain the elemental compositions of the oxide thin films. 2-MeV 4He^+^ ions were used for the RBS measurements, in which backscattered ions were detected at a scattering angle of 170°. The possible ion-channeling effects in both substrates and thin films were minimized by adjusting the incidence angle to 5° with respect to the surface normal and performing multiple-small-random-angular movements within a range of 2° during data acquisition. SIMNRA (*67*), version 7.02, was used for simulating the RBS spectra and determining the elemental compositions of the films. For all measured compositions, the maximum systematic uncertainty arising from the effect of stopping cross sections is <0.8%, and the maximum statistic uncertainty from the number of experimental counts is <1.1%.

### Time-of-flight elastic recoil detection analysis

In these measurements, recoils were detected at an angle of 45° with respect to the incident beam in a telescope that measured ToF, using a foil-detector, and energy in a gas ionization chamber in coincidence. This approach results in mass resolved data in ToF-versus-Energy plots. Recoils were created using a 44-MeV ^127^*I*^8+^ beam incident at 67.5° with respect to the sample surface normal. ERDA was carried out using the same 5-MV 15SDH-2 tandem accelerator as used for RBS.

### Depth-resolved XPS

The surface chemistry (chemical bonding and oxidation states) evolution of the oxide layers was analyzed as a function of depth by XPS in a Kratos Axis Ultra DLD instrument. To avoid the destructive effects of Ar^+^ ion sputter etching on XPS core levels (*49*), the surface of the oxide thin films was capped in situ with a few-nanometer-thick TiN layer in the same PLD system as the film deposition before air exposure. High–energy resolution core level XPS spectra were acquired using monochromatic AlK_α_ radiation (*hν* = 1486.6 eV). Sputter etching was carried out using a 0.5 keV Ar^+^ ion beam incident at 70° with respect to the sample normal. The analyzed sample areas were 0.3 mm by 0.7 mm, located in the center of 0.3 mm by 0.7 mm sputter-etched regions. The binding energy scales were calibrated against the Fermi edge recorded from the sputter-etched layers to avoid uncertainties arising from using the C 1s peak from adventitious carbon (*68*). Hf 4f XPS core-level spectra were deconvoluted with the CasaXPS software (www.casaxps.com/) and the accuracy of the de-convolutions was ensured by maintaining the same line shapes, 4f7/2-4f5/2 binding energy separation, peak-to-area ratio, and full width at half maximum values, while varying the peak areas and positions. As the thin films (15 nm) were in electrical contact with the sample holder through the conductive substrate, any peak shifts are unlikely to occur because of sample charging, which is typically observed for insulating samples without proper electric contact to the sample holder. The backgrounds of all spectra were subtracted using the Shirley approach (*69*), and quantification was performed by using elemental sensitivity factors provided by Kratos Analytical Ltd. (CasaXPS_KratosAxis-F1s).

### UV photoelectron spectroscopy

UPS measurements were performed at the He-I line (21.21 eV) using a SPECS UVS 10/35 differentially pumped capillary discharge vacuum UV source in an ultrahigh vacuum chamber. The system work function was calibrated using clean Ag and Au foils. A bare Nb:STO substrate and a Ba:HfO*_x_* film on Nb:STO were annealed in vacuum at 240°C to remove carbon contaminates and then vacuum-transferred to the UPS chamber. High-resolution scans were measured around the secondary electron cutoff, and the valence band maxima (VBM) for the two samples were determined by line fits, which yielded an SE cutoff of 16.92 ± 0.02 eV and 16.98 ± 0.02 eV for Nb:STO and Ba:HfO*_x_* respectively, indicating a vacuum offset of −0.06 ± 0.04 eV. Similarly, the VBM were determined to be 2.98 ± 0.2 eV for Nb:STO and 3.49 ± 0.2 eV for Ba:HfO*_x_*. With the Fermi edge calibrated as 0 eV for Nb:STO by the Ag and Au references, the substrate work function Φ is calculated to be 4.29 eV (Φ = *hν* − *E*_cutoff_ − *E*_Fermi_), well aligned with literature values (*70*).

## Supplementary Materials

### This PDF file includes:

Supplementary Text Figs. S1 to S21 Table S1 References
