# Supplementary material for: Thin film design of amorphous hafnium oxide nanocomposites enabling strong interfacial resistive switching uniformity
Source: Sci Adv. 2023 Jun 21;9(25):eadg1946. doi: 10.1126/sciadv.adg1946 (PMC10284547; doi:10.1126/sciadv.adg1946)
Supplement: Supplementary file 1 — Supplementary Text Figs. S1 to S21 Table S1 References [file sciadv.adg1946_sm.pdf]

Science Advances
AAAS

Supplementary Materials for

Thin film design of amorphous hafnium oxide nanocomposites enabling strong interfacial resistive switching uniformity

Markus Hellenbrand *et al.*

Corresponding author: Markus Hellenbrand, mkhh2@cam.ac.uk; Judith L. MacManus-Driscoll, jld35@cam.ac.uk

Sci. Adv. **9**, eadg1946 (2023)
DOI: 10.1126/sciadv.adg1946

This PDF file includes:

Supplementary Text
Figs. S1 to S21
Table S1
References

Reference Pt on Nb:STO

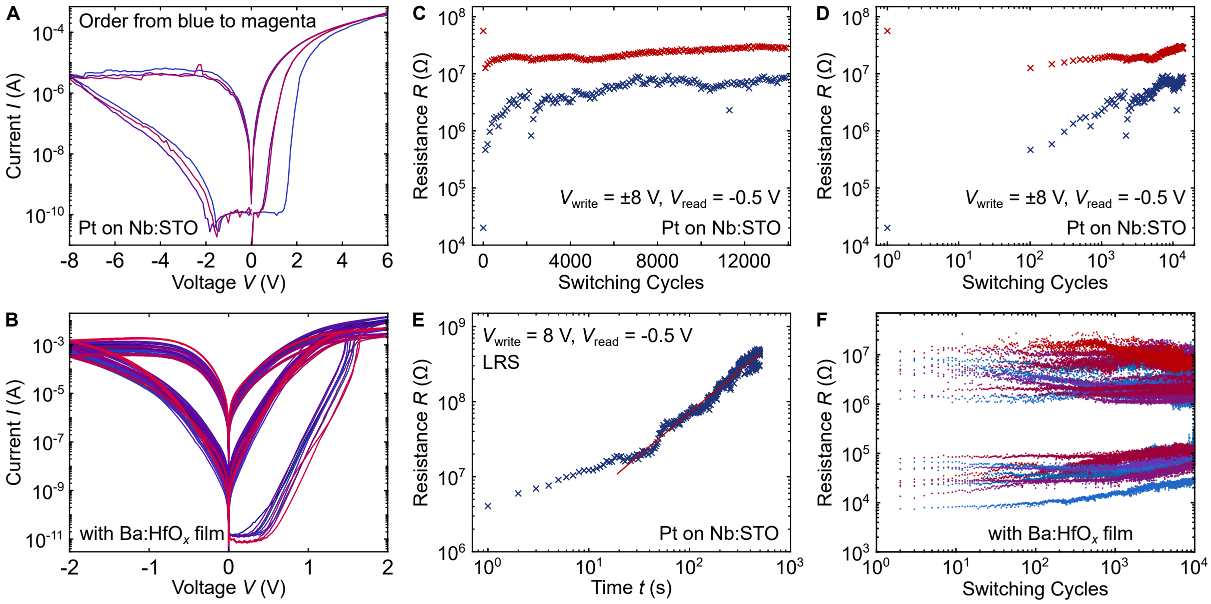

Figure S1: Reference measurement of Pt electrodes deposited directly onto Nb:STO. (**A**) IV curves, three cycles on the same device, representative of the overall sample behavior. Note especially the different shape of the curves and the large difference in voltages when compared with the devices with Ba:HfO$_x$ films. (**B**) Same figure as in the main manuscript for ease of comparison. Ten devices, one color per device, five repetitions for each device. It is clear that the shape and uniformity of the *IV* curves as well as the voltage range required for resistive switching change substantially when inserting the Ba:HfO$_x$ nanocomposite film between TE and the Nb:STO substrate. (**C**) & (**D**) Endurance for Pt on Nb:STO with a linear and logarithmic x-axis, respectively. After a rapid collapse of an initially large memory window, this material stack cannot maintain a memory window >10 beyond a few 100 cycles. (**E**) Retention of the LRS. After the first few seconds, the decay follows a power law $R = A \times t^{\beta}$, the Curie-von Schweidler law, similar to the decay of the devices with a thin film in between. For Pt on Nb:STO, it is established that charge trapping (*25*) or proton exchange (*26*) are the major origins of hysteretic *IV* curves, but a decay due to the Curie-von Schweidler law in general only indicates a time-dependent charge redistribution in a dielectric (*31*) and can just as well be due to ionic migration. The differing slope at lower times t can be attributed to a secondary effect with different time constants. (**F**) Same endurance data as in the main manuscript (for devices with Ba:HfO$_x$ thin films), but with a logarithmic x-axis.

Histograms for endurance measurements

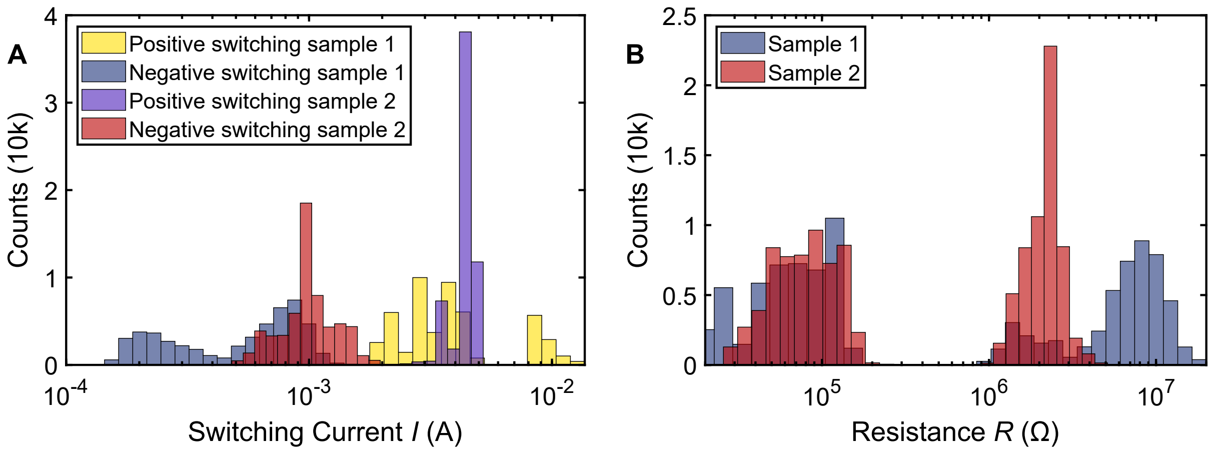

Figure S2: Histograms for the measured switching endurance summarized in main text Fig. 1B. (**A**) Positive and negative switching currents. Yellow and blue: sample 1, purple and red: sample 2. While for sample 2, the positive and negative switching currents are limited to one distribution each, sample 1 displays two bimodal distributions, which reveals that the variation among devices on this sample appears in two 'classes' of devices. (**B**) Resistances of high and low resistance states. Blue: Sample 1, red: sample 2. Corresponding to the two bimodal distributions of switching currents in (A), the HRS of sample 1 displays a bimodal distribution of resistance states. When comparing with (A), a similar bimodal distribution is discernible from the two bins with the lowest resistances as a consequence of the bimodal switching current distribution. While the HRS and LRS each stretch over about an order of magnitude, they are also separated by about an order of magnitude, resulting in a constant minimum memory window.

IV curves for samples with different electrodes

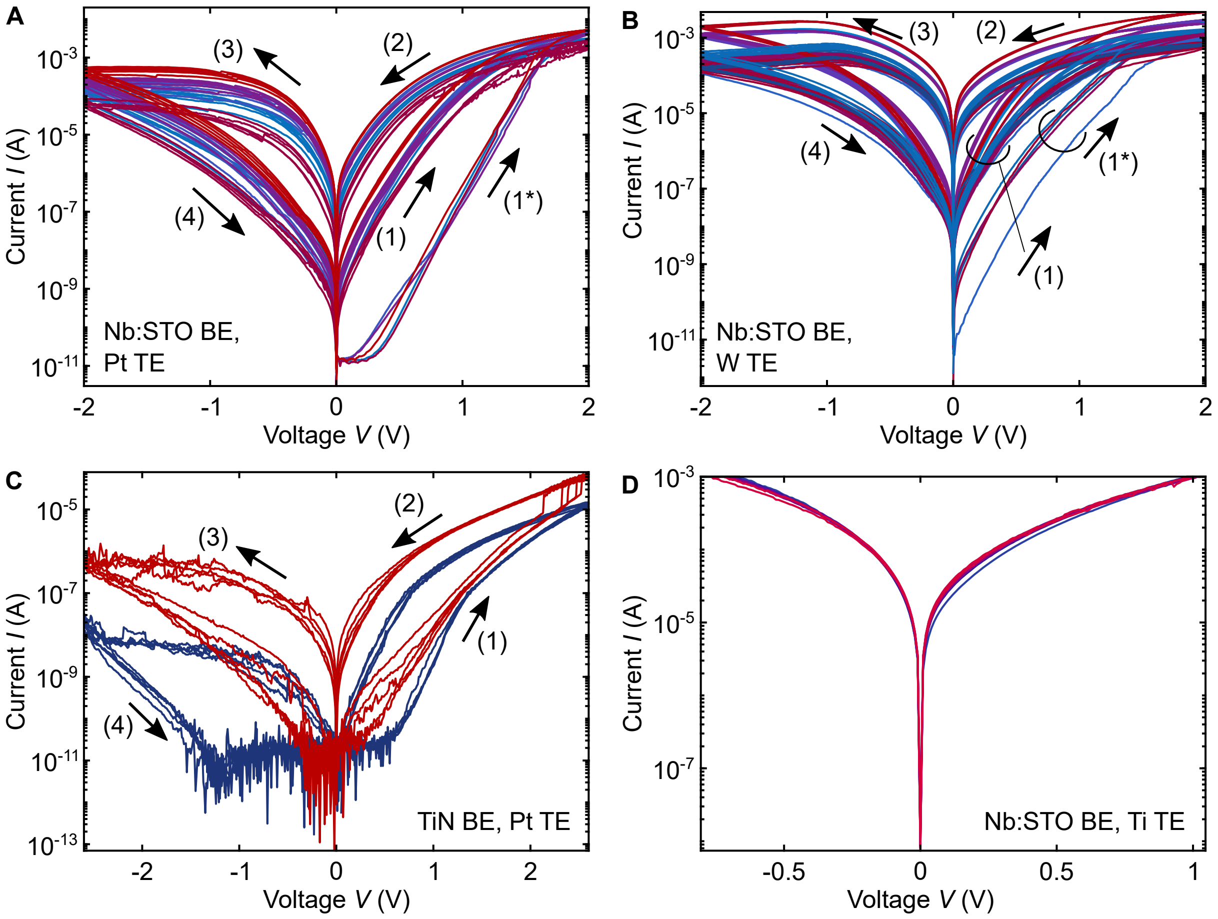

Figure S3: IV curves for samples with different electrodes. Different colors correspond to different devices, five repetitions per color, numbered arrows indicate the switching polarity, where (1*) indicates the forming step. (**A**) A second sample with a Pt-Ba:HfO_x-Nb:STO stack demonstrating good device-to-device and sample-to-sample reproducibility. The *IV* curves have the same shape and similar uniformity as the sample in the manuscript. (**B**) A sample with a W-Ba:HfO_x-Nb:STO stack demonstrating that Pt is not critical as the top electrode. Despite the different metal work functions, similar *IV* curve shapes and uniformity are achieved as on the samples with Pt top electrodes. This sample with W TE was used for all neuromorphic demonstrations. (**C**) Two *IV* curve shapes on the same device based on a Pt-Ba:HfO_x-TiN stack, where the TiN was deposited by pulsed laser deposition on an insulating MgO substrate. The blue curves are the initial five sweeps, the red curves are after a retention measurement. While the curves are more noisy and change over time, they demonstrate that a similar hysteresis can be achieved without an Nb:STO substrate. The lack of uniformity and stability can be

attributed to the TiN not having been optimized, and better uniformity can be expected after the respective optimization. The small rapid current increases at the highest positive voltages for the red curves support the assumption of the involvement of a (partial) filament in addition to the actually dominant resistive switching process. (**D**) A device with a Ti top electrode. No resistive switching could be achieved, and the devices are very conductive. Most likely, the oxide films become so conductive because the Ti scavenges a lot of oxygen from them.

Comparison of nanocomposite and pure HfO_x films

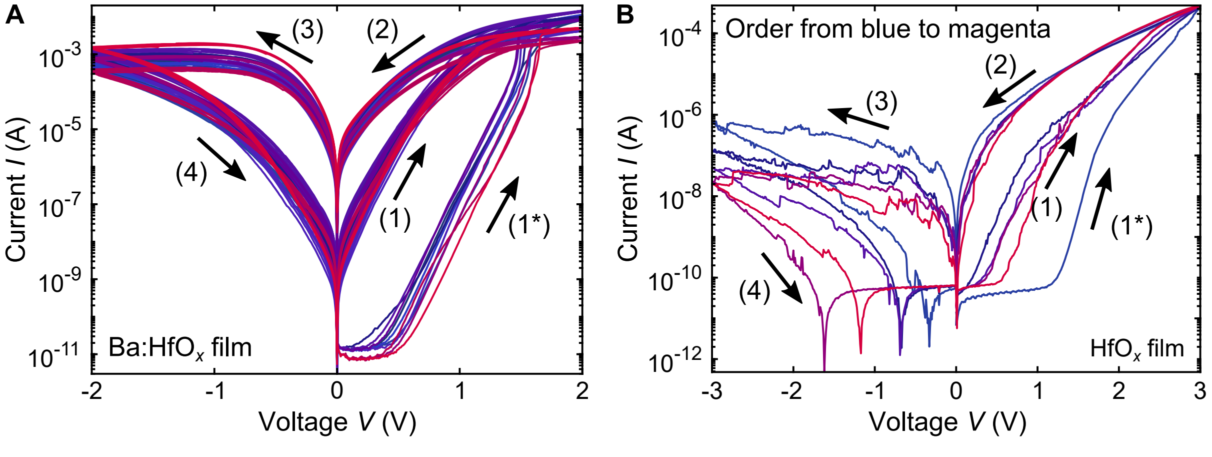

Figure S4: Comparison of nanocomposite and pure HfO_x films. (**A**) Ba:HfO_x films (same as main Fig. 1A for convenience of comparison) and (**B**) pure HfO_x films (single device, five *IV* repetitions, different color for each repetition). Numbered arrows indicate the switching polarity, where (1*) indicates the forming step. The pure HfO_x films are less conductive, and even within the same device, the *IV* curves are less stable than with the Ba:HfO_x films.

Endurance and retention for devices with 20 µm diameter

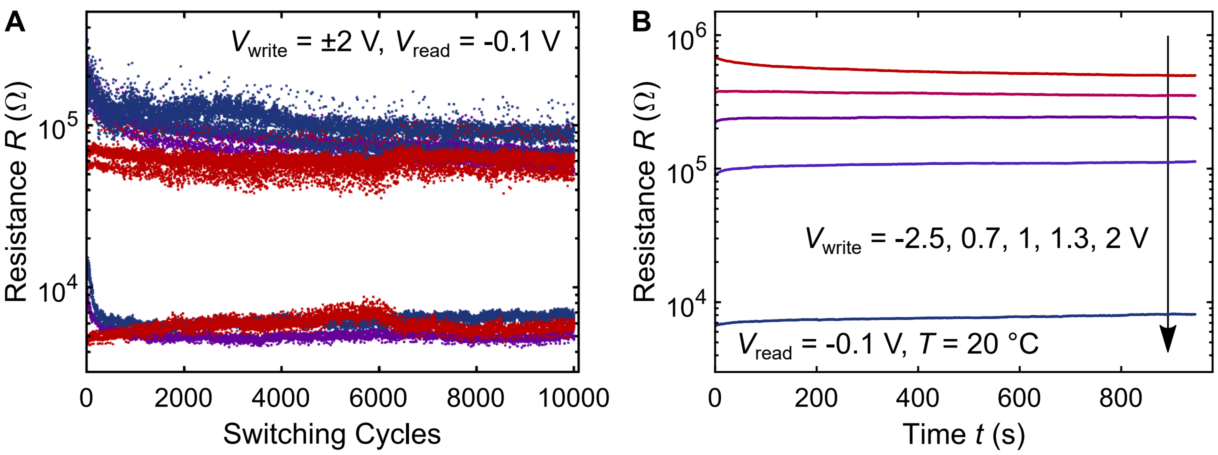

Figure S5: Endurance and retention for devices with 20 µm diameter. Example data for (**A**) the switching endurance of three different devices with 20 µm top electrode diameter and (**B**) multi-level state retention of one of these devices. (A) While two of the devices undergo some form of 'endurance relaxation' during the first switching cycles, the endurance performance is not degraded compared with the larger devices presented in the main text Fig. 1B. Note, that the devices here have W top electrodes and these devices had higher off-state currents than the Pt devices (see Fig. S3A & B). As a consequence, the absolute resistance values for the endurance measurement are lower than the ones in the endurance Fig. 1B, but the memory window is similar at ≈ 10. (B) This also applies to the multi-level state retention when compared with the larger devices presented in main text Fig. 2 as again, especially the resistance in the HRS is lower in the W devices here, but no performance degradation is observed with respect to the number of resistance levels, their spacing, or retention. (Further for a fair comparison, note that the data here was measured at room temperature, whereas the data presented in Fig. 2 were measured at $T = 85$ °C. Thus, the data here looks more stable than in Fig. 2.)

Conductive atomic force microscopy (CAFM) and topography scans

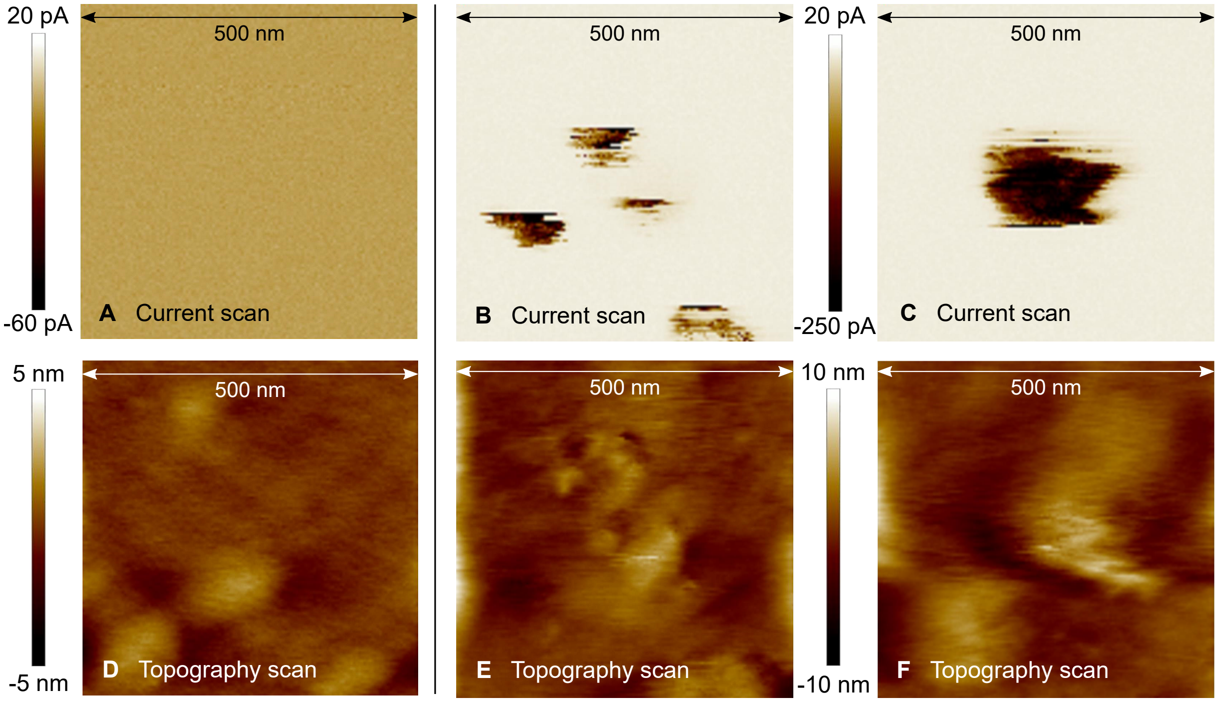

Figure S6: CAFM scans of a bare Ba:HfO$_x$ film deposited at 400 °C. (**A**)–(**C**) Current scans, (**D**)–(**F**) corresponding topography scans, recorded simultaneously. (A) For the pristine film, the root mean square surface roughness is <1 nm. (B) The same area as (A) after a scan with -10 V applied to the substrate. Small conductive areas formed in parts of the film. (C) In a controlled version of (B), but a different area, the center area was scanned a few times with -10 V applied to the substrate to make it conductive ('forming'). The larger scan in (C) with -7 V applied to the substrate clearly shows the difference between the formed center area and the insulating area surrounding it. Note that in CAFM, larger voltages are required than in a probe station measurement due to the large contact resistance between the CAFM tip and the film, as well as due to current crowding effects.

Fast switching measurements

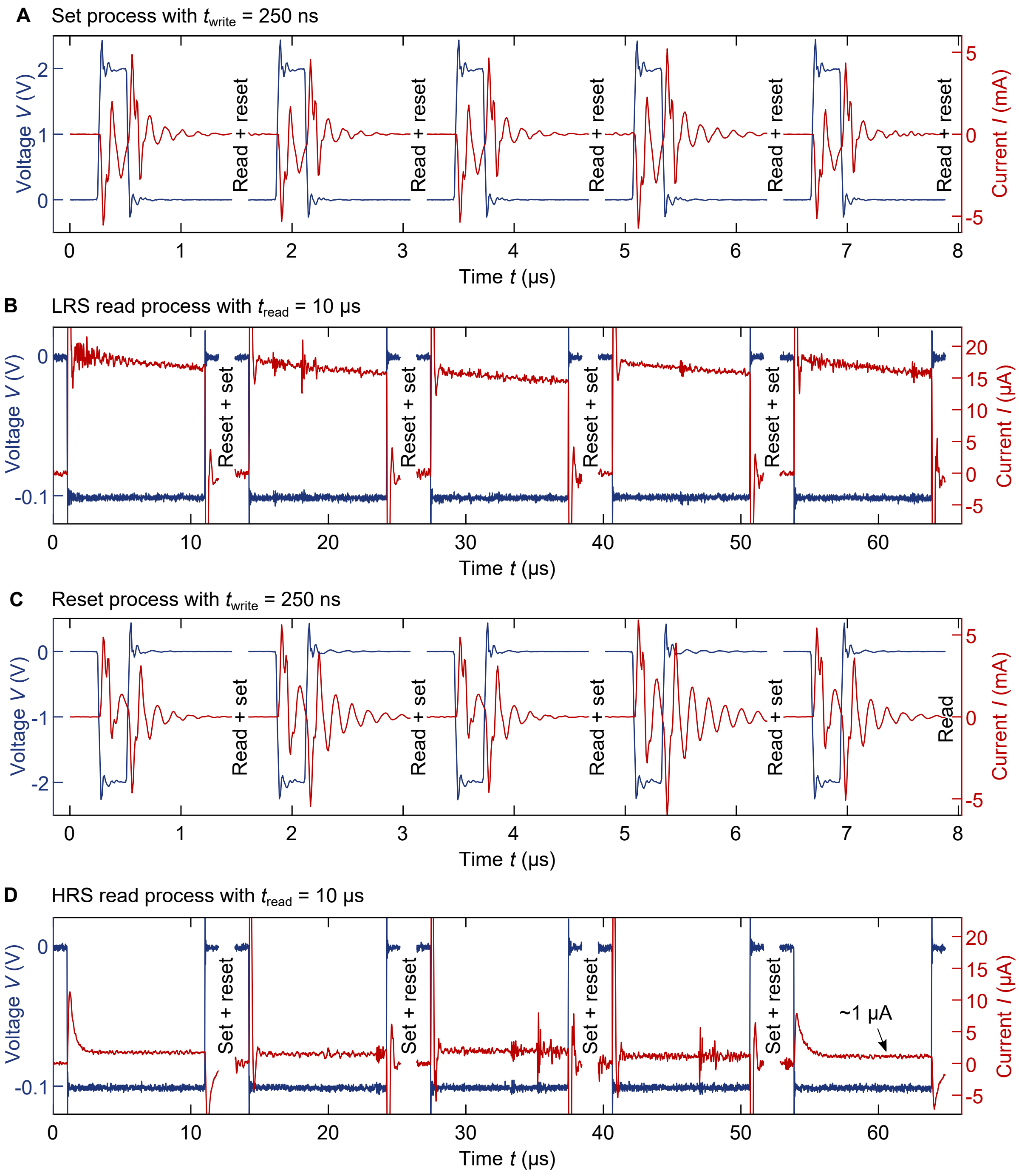

Figure S7: Real-time data of fast switching measurements. Voltages (blue, left y-axis) and currents (red, right y-axis) are presented as a function of time during fast switching measure-

ments, here exemplary with 250 ns switching pulse widths with 10 ns rise times. (20 ns example to follow below.) Note that the four respective operations (set, read, reset, read) are summarized in a single figure each (e.g. all set operations in one figure), but the processes were carried out in the 'usual' order, i.e. set, then read, then reset, then read. (**A**) & (**C**) Voltages and currents during switching pulses, (A) for the set operation, (B) for reset. It is clear that the fast voltage rise time causes a strong current ringing due to RC time constants in the signal path and the actual switching current cannot be observed. Dedicated high-frequency probe pads and ground-signal-ground probe tips would be required for this. (**B**) & (**D**) Separate read pulses with 10 µs widths evidence that the devices switch between LRS and HRS regardless of the ringing. As the voltage signal suffers from much less ringing and the devices switch at much larger programming pulses, too ($\sim$ms in main text Fig. 1), it is also clear that the ringing is not in fact required for the devices to switch. For 'clean' real-time current-vs-time measurements, dedicated probe pads and tips are required, as demonstrated in e.g. (*17*), but the development of a corresponding process was beyond this study.

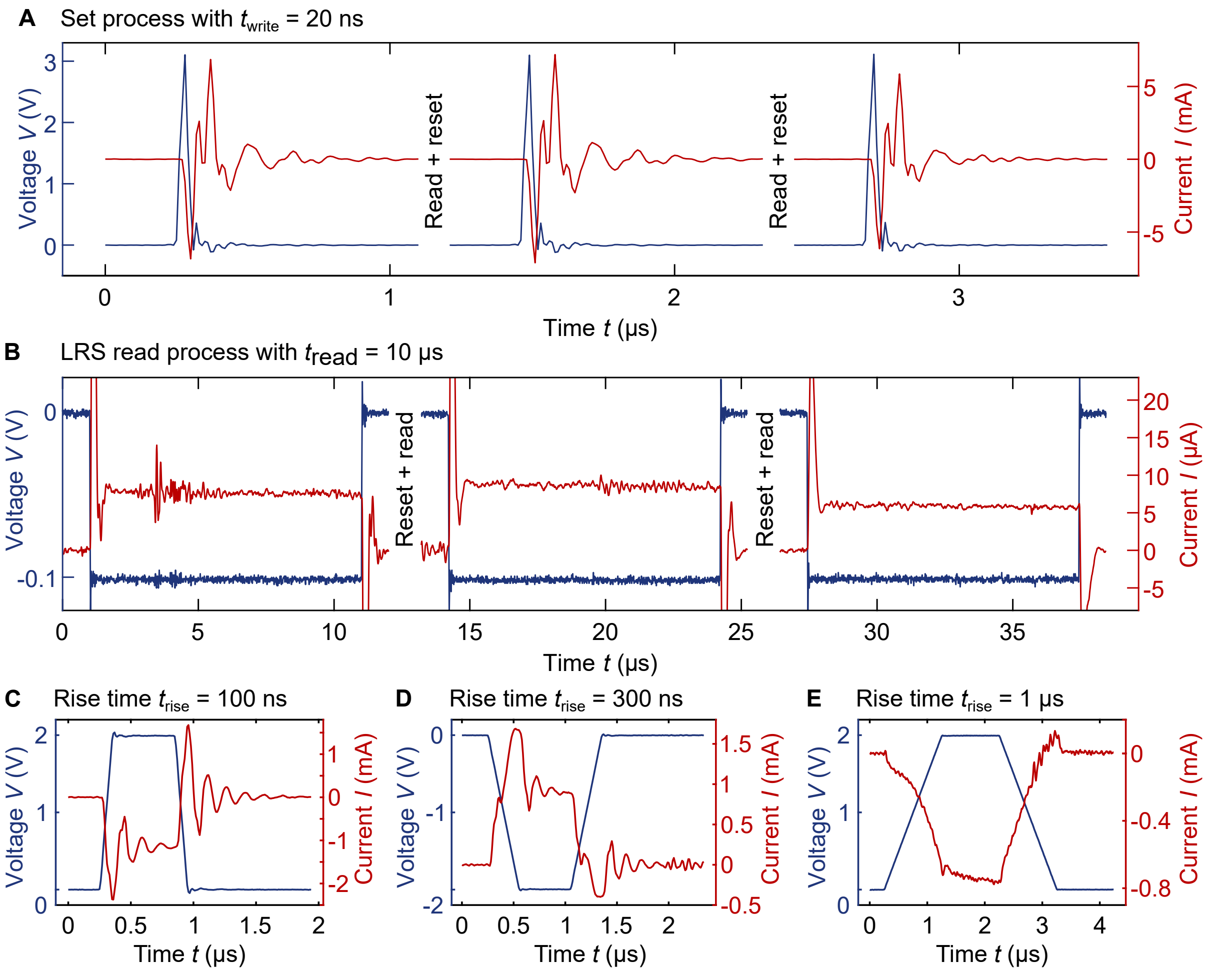

Figure S8: Real-time data of fast switching measurements, continued. (**A**) & (**B**) Voltages (blue, left y-axis) and currents (red, right y-axis) for 20 ns switching (A) and subsequent read operations (B). (A) Note that the nominal set voltage was still 2 V, but the increased ringing at these short times lead to a more pronounced voltage overshoot. In between the three presented pulses, the state was read out as presented in (B) and the device was reset. (B) When compared with the read operations in Fig. S7B, it is clear that now the memory window is considerably smaller. (**C**)–(**E**) Increasing switching pulse rise times from 100 ns to 1 µs. With increasing rise times, the current ringing decreases until it subsides for 1 µs rise times, when the current follows the voltage instantaneously.

Temperature-dependent state retention

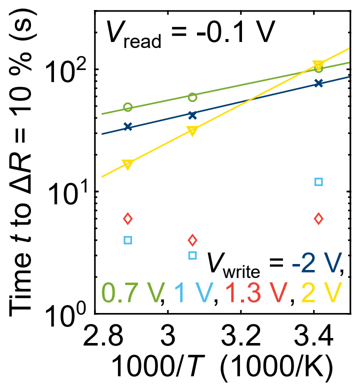

Figure S9: Temperature-dependent measurements of time to 10% state decay. Further to the investigations of the resistance state degradation in Fig. 2, this figure presents the time t until the initially set resistance R had changed by 10%, measured at different temperatures T for different resistance states in the same device (note, however, different device from Fig. S9). For this type of characterization, it is typically assumed that at higher temperatures, any degradation process is accelerated due to the higher thermal energy at the disposal of the atoms in the device, and often, this can be modeled with and Arrhenius-type equation $\propto \exp[-E_a/(k_B T)]$ with an activation energy E_a and the Boltzmann constant k_B. Here, in the measured temperature range, only some of the measured states decayed according to such a dependence, but for example, an extrapolation of the LRS with $V_{write} = 2\,V$ indicates that a 90% state retention for 10 years would require a temperature as low as 132 K. While this might be useful for cryogenic memory applications such as space or quantum computing applications, it not sufficiently non-volatile for conventional long-term data storage.

Devices based on films deposited at 800 °C

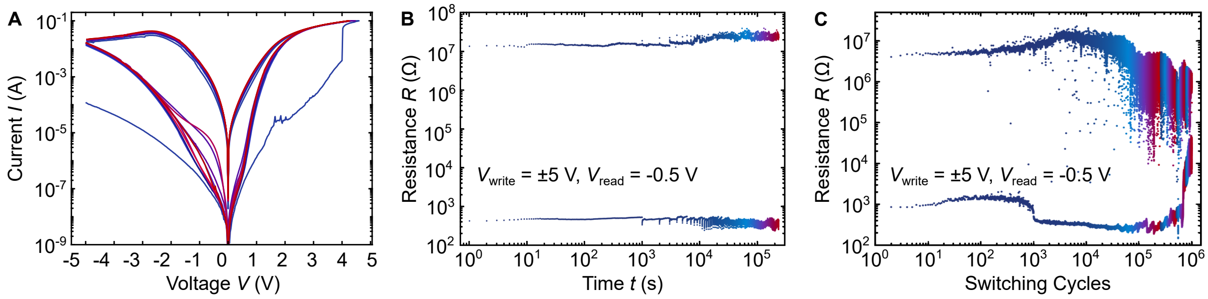

Figure S10: *IV* curve, retention, and endurance examples from devices made from Ba:HfO$_x$ thin films deposited at 800 °C. (**A**) Initial ten *IV* curves for a pristine device, order from blue to red. As it required ± 5 V to form the device, ± 5 V was adopted for the retention and endurance measurements, too. The only reason why the *IV* cycling started at -5 V instead of 0 V was that the later standard cycling protocol had not been implemented yet. (**B**) Retention of a memory window $>10^4$ without any sign of degradation for $>10^5$ s. The small bumps are a result of intermittent data transfers between the measurement instrument and the computer; no re-programming occurred at these points. (**C**) Endurance measurement for the same device. While the initial 1000 cycles are stable at a memory window $>10^3$, the device degrades beyond that, first the HRS, later also the LRS.

As the switching currents at ± 5 V are about an order of magnitude higher than in the devices presented as the main results, and there is a characteristic rapid current increase during forming, it is likely that in these devices with films deposited at 800 °C the switching mechanism is filamentary. This would also explain the excellent retention properties.

X-ray diffraction spectra

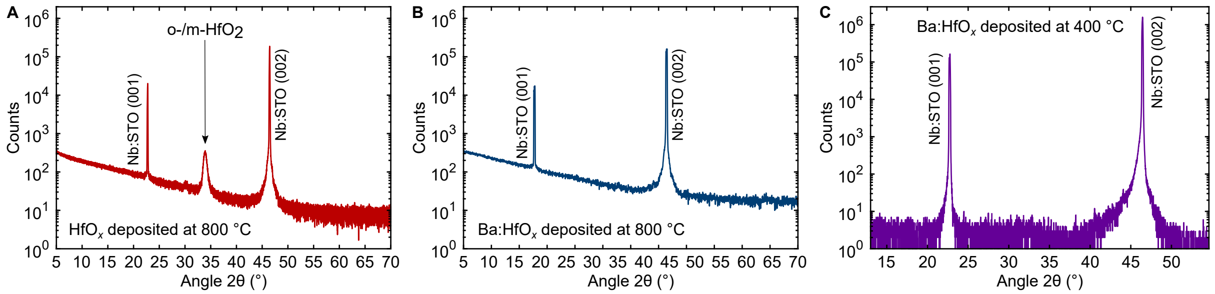

Figure S11: X-ray diffraction spectra for different thin films deposited at different temperatures. (**A**) Pure HfO_x deposited at 800 °C –– the peak at 34 ° can be identified as monoclinic $(0\,0\,2)$, $(0\,0\,\bar{2})$, $(2\,0\,0)$, $(\bar{2}\,0\,0)$, or orthorhombic $(0\,0\,2)$, $(0\,0\,\bar{2})$. As HfO_x preferentially grows in the monoclinic phase, this is the more likely orientation. (**B**) In the Ba:HfO_x nanocomposite films deposited at 800 °C, there is no indication of any long-range crystallinity even at the deposition temperature of 800 °C. The Ba clearly suppresses the formation of crystallites even at high deposition temperatures. (**C**) The same observation holds for Ba:HfO_x composite films deposited at 400 °C; there is no sign of any long-range crystallinity. The different noise floors and intensities are a result of having had to use different detectors for the measurements. (A) & (B) were measured with a PIXcel3D position sensitive detector, whereas (C) was measured with a single-point proportional detector.

TEM 800 °C films

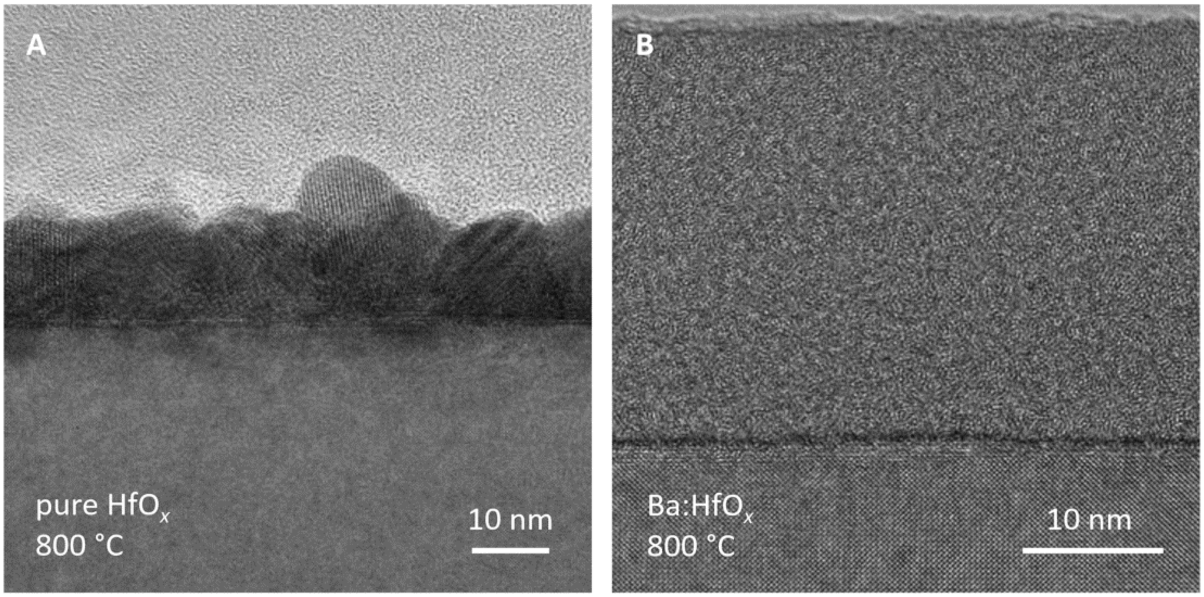

Figure S12: Transmission electron micrographs of reference films deposited at 800 °C. (**A**) Pure HfO_x with clear signs of polycrystallinity. Curiously, the films are less well oriented than the ones deposited at 400 °C. (**B**) The addition of Ba completely suppresses the formation of crystallites and makes the films amorphous or possibly very-short-range-order nanocrystalline. These TEM scans correspond to the films in Fig. S11A & B.

Rutherford backscattering spectrometry

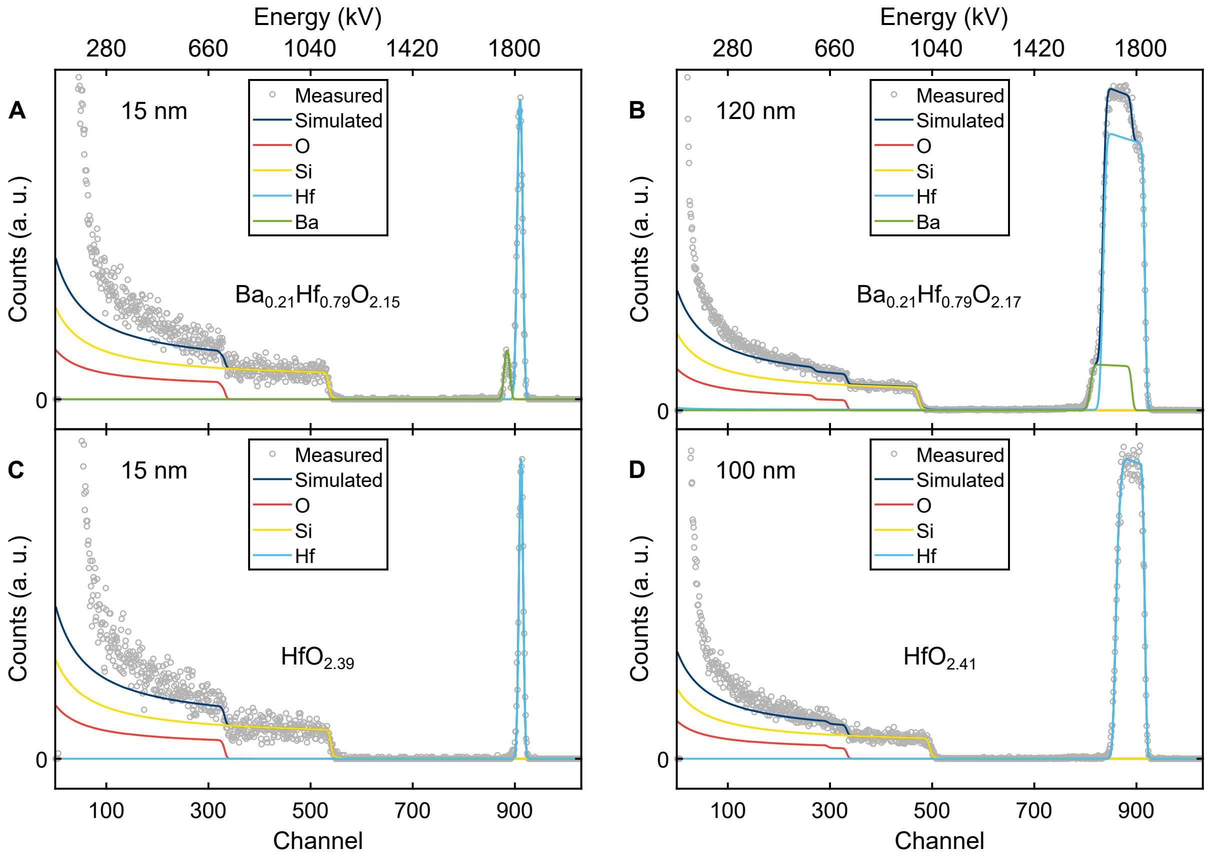

Figure S13: Rutherford backscattering spectrometry to measure the total composition of the nanocomposite and reference thin films deposited at 400 °C. In all plots, grey circles correspond to the measured spectra and colored lines visualize the compositions calculated to match the experimental results. SIMNRA (*67*), version 7.02, was used for the calculated results. The total film compositions are provided in the figures. (**A**) & (**B**) 15-nm- and 120-nm-thick Ba:HfO_x composite films. (**C**) & (**D**) 15-nm- and 100-nm-thick pure hafnium films. The large measured signal at low energies/channels is due to increasing amounts of inelastic scattering in the substrate as opposed to the elastic scattering, which RBS modeling is based on. To minimize any uncertainties related to the low-energy oxygen signal, the RBS results were corroborated with the time-of-flight elastic recoil detection analysis below. The modeled atomic fractions based on the measurements in Fig. S13 are listed in Table S1 below.

The Ba fractions are in agreement with the EDX result of Fig. 4F in the main manuscript.

Table S1: RBS results, values in atomic percentage; compositions $Hf_{1-x}Ba_xO_y$ with x = Ba/(Hf + Ba) and y = O/(Hf + Ba).

Sample	Ba	Hf	O	Composition
Pure HfO_x 100 nm	0	29.3	70.7	$HfO_{2.41}$
Pure HfO_x 15 nm	0	29.5	70.5	$HfO_{2.39}$
Ba:HfO_x 120 nm	6.7	24.8	68.5	$Ba_{0.21}Hf_{0.79}O_{2.17}$
Ba:HfO_x 15 nm	6.7	25	68.3	$Ba_{0.21}Hf_{0.79}O_{2.15}$

Depth-resolved XPS

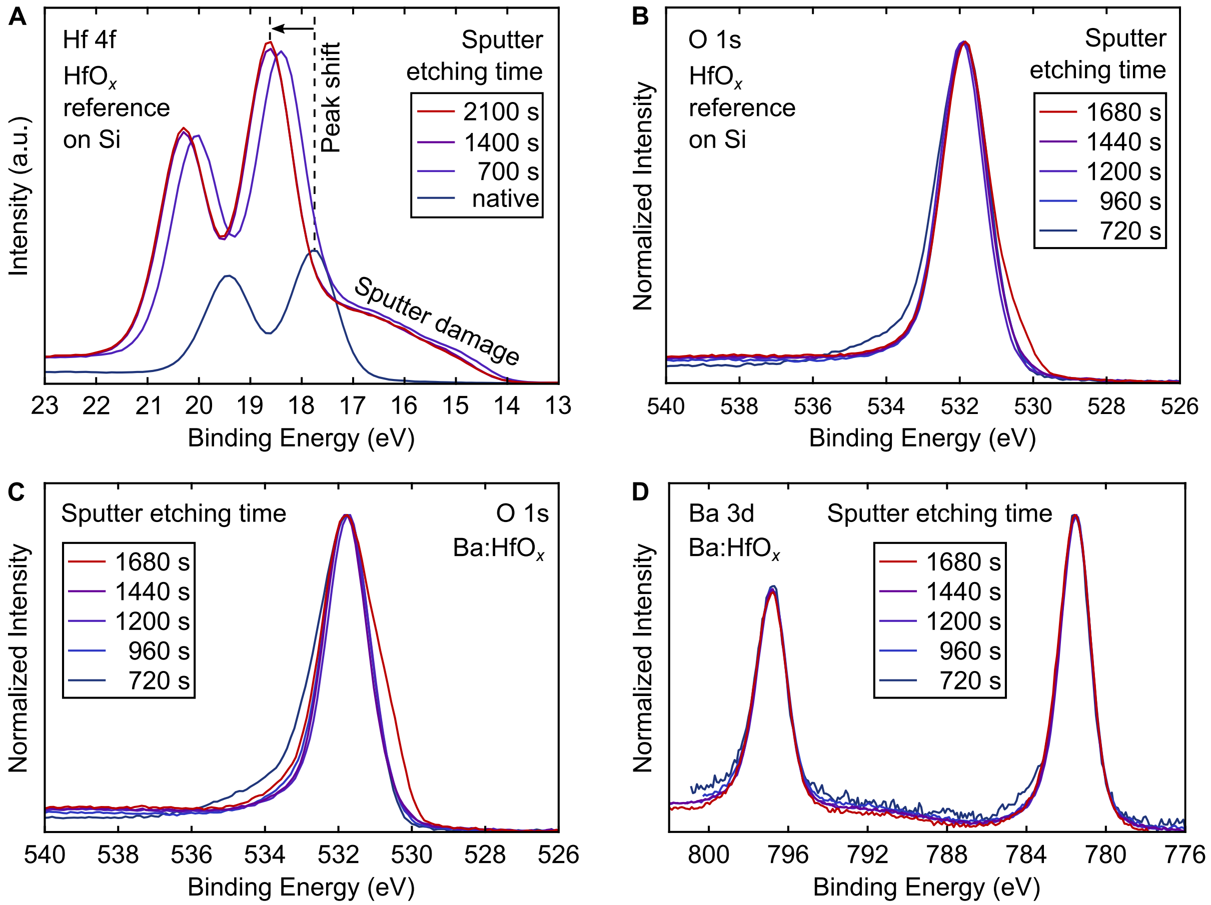

Figure S14: Core spectra for depth-resolved XPS, all films deposited at 400 °C. (**A**) Hf 4f reference core spectra for HfO_x deposited on Si to reveal the effect of Ar ion sputter damage. For these reference measurements, ~ 30 nm of pure HfO_x were deposited with the same deposition parameters as the RS devices (except longer deposition time and no TiN capping) on a Si substrate. As presented in Fig. S14A, the experiment confirmed that sputter etching results in a peak shift, peak broadening, and the formation of a broad shoulder in the lower-binding-energy range of the Hf 4f spectra. However, different from the measurements presented in Fig. 5C & D in the main document, in these reference Hf 4f core level spectra for $t > 700$ s the shape of and area below the low-energy shoulder remain the same with increasing sputter time. This proves that the gradual increase of the shoulder in Fig. 5C & D is not an artifact from sputter damage but inherent to the films. The increasing shoulder thus reveals a significant change of the concentration of Hf sub-oxidation states as a function of depth towards the Nb:STO substrate. However, as there are no reliable references available for the peak modeling of such features

which are intermixed with the influence of sputter damage, this part of the Hf 4f spectra was not de-convoluted and instead fitted with only one broad peak comprising both Hf^{2+} and Hf^{1+} components. It is pointed out that the Hf 4f spectra of neither sample contained any metallic Hf^{0} component, which would have appeared at ≈ 14 eV.

Fig. S14 (**B**) & (**C**): O 1s core spectra for HfO_x reference (on Nb:STO) and Ba:HfO_x composite film. There is no significant change with increasing sputter time. The high- and low-energy shoulder can be attributed to O-N bonds to the protective TiN capping layer and oxygen from the substrate, respectively. (**D**) Ba 3d core spectra for a Ba:HfO_x sample. No significant change occurs with increasing sputter time.

XPS fitting

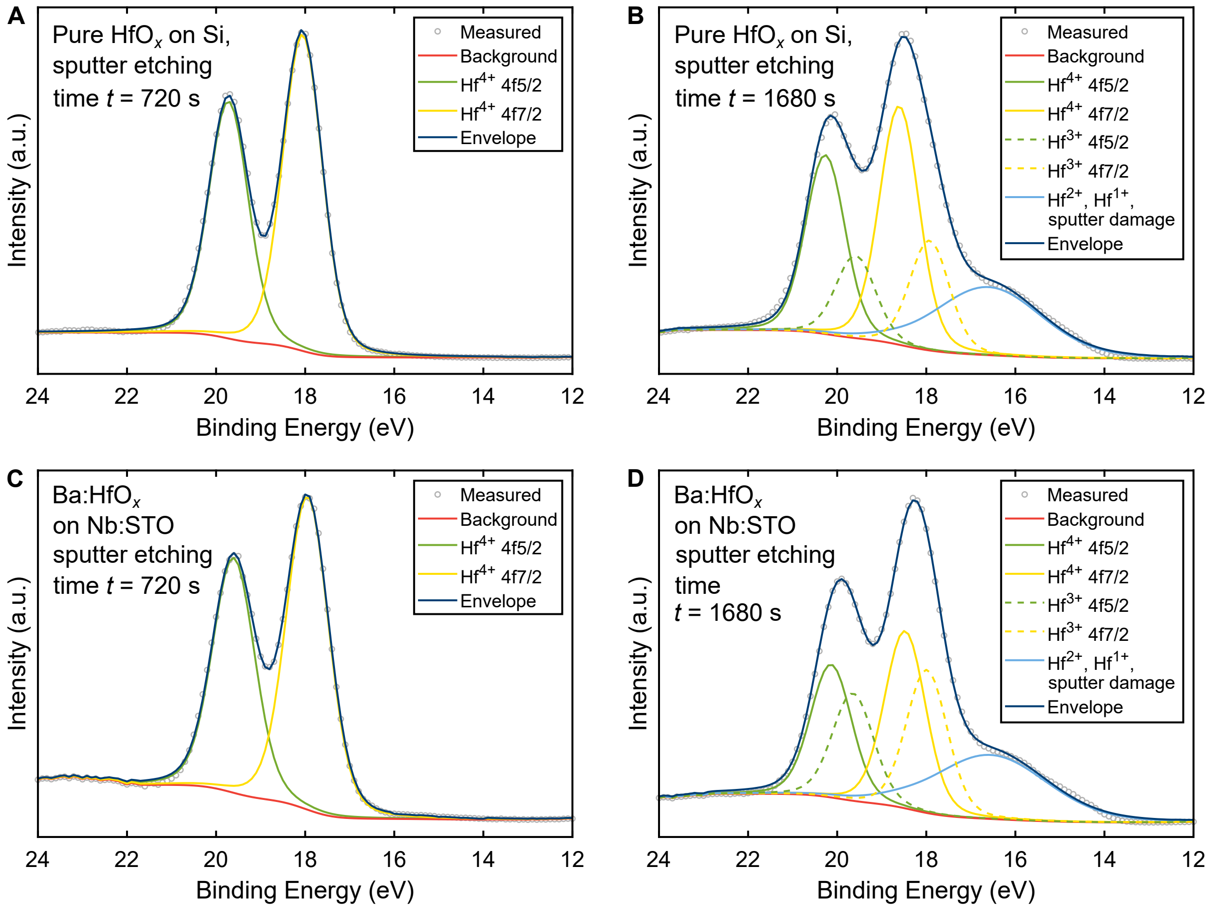

Figure S15: Examples of how the XPS data was fitted. (**A**) & (**B**) Hf 4f core spectra of a pure HfO_x reference film after 720 s and 1680 s, respectively. (**C**) & (**D**) Hf 4f core spectra of a Ba:HfO_x nanocomposite film after 720 s and 1680 s, respectively.

For accurate fitting results, first, the single doublet pairs in (A) and (C) were fitted. Then, for accurate deconvolution of the peaks for all following etching times, the spectra were fitted using the same line shape, the same peak to area ratio, a constant distance of 0.6 eV between the 4f5/2 and 4f7/2 peaks for Hf^{4+} and of 0.4 eV for Hf^{3+}, a constant area ratio of 75% between 4f5/2 and 4f7/2 both for Hf^{4+} and Hf^{3+}, and constant values for the full width at half maximum. for all sputter etch times.

(A) & (C) Pure HfO_x and $BaHfO_x$ composite film, respectively, after 720 s of etching. This etching time removed most of the TiN capping layer, but without damaging the actual films yet, so that the observed peaks do not contain any sign of sputter damage. For both films, the two

peaks can be fitted well with single doublet pairs of 4f7/2 and 4f5/2 states corresponding to fully oxidized Hf, i.e. Hf^{4+}.

(B) & (D) Same films after 1680 s of etching. A clear peak broadening is observed, so that now, the 4f7/2 and 4f5/2 peaks can only be fitted by spin-split 4f7/2-4f5/2 doublet states, which reveals the additional presence of Hf^{3+}. Both Hf^{3+} and Hf^{4+} are shown in the figures. In addition, the shoulder corresponding to Hf^{2+} and Hf^{1+} is clearly visible. As discussed above (Fig. S14), a constant contribution to this shoulder results from sputter damage to the films, but as is evident from he progressively increasing shoulder presented in the main manuscript (Fig. 5), the remaining part is due to an actual change in the film composition. However, as mentioned before, as there are no reliable references available for the peak modeling of such features which are intermixed with the influence of sputter damage, this part of the Hf 4f spectra was not de-convoluted and instead fitted with only one broad peak comprising both Hf^{2+} and Hf^{1+} components.

Area dependence

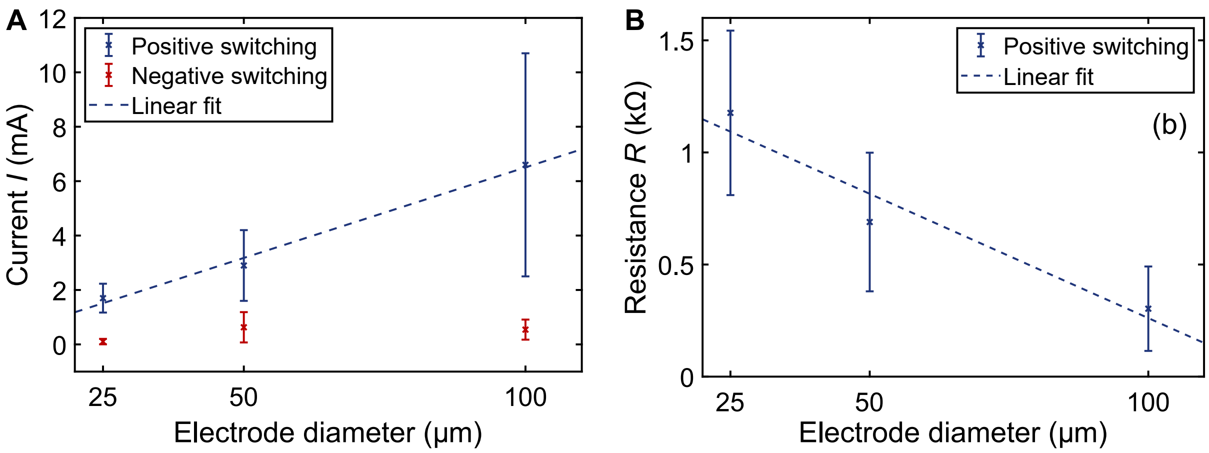

Figure S16: Area scaling of the switching currents of the Ba:HfO$_x$ nanocomposite devices. (**A**) Switching currents of the Ba:HfO$_x$ *IV* curves, i.e. the currents at ± 2 V, and (**B**) the corresponding resistances at +2 V. (A) Blue, +2 V: Despite the increasing error bars for the measured currents, a linear function of the electrode diameter can be fitted to the average values. The currents increase by a factor ≈ 2 when the electrode diameter is doubled. Red, -2 V: No obvious dependence of the high resistance state on the electrode diameter. (B) Resistances corresponding to the positive switching currents in (A) with a linear fit. The (higher) resistances corresponding to the negative switching are not plotted so as not to distort the y-axis and thus render the linear fit for the positive switching resistances indiscernible. (Also, as pointed out, no linear trend is observed for the negative switching.) Due to the linear scale, the error bars appear larger than the variation in Fig. 1A with a logarithmic y-axis suggests.

Error bars represent the standard deviation of the measurement of different devices; five devices each for 25 and 50 μm diameters, ten devices for 100 μm diameter. The linear scaling of the current with the electrode diameter can be explained (qualitatively) by the observation of different lengths of the Ba-rich columns in Fig. 4. With a certain lengths distribution where not all columns contribute to the device current equally, the current can scale with the diameter instead of the area.

Temperature dependence

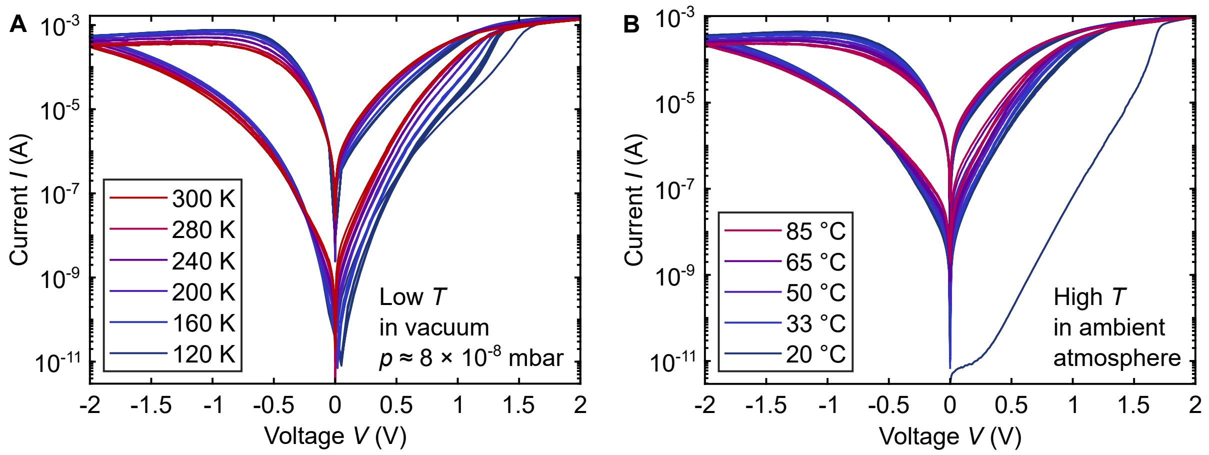

Figure S17: Temperature-dependent *IV* measurements of two different devices based on Ba:HfO$_x$ nanocomposite films. Films deposited on Nb:STO at 400 °C ; Pt top electrodes. (**A**) Temperatures varied from 120 K to 300 K as indicated in the legend and the measurements were carried out in a vacuum of $p \approx 8 \times 10^{-8}$ mbar. Three *IV* cycles per temperature. The device had been formed before the temperature-dependent measurements. (**B**) Temperatures varied from 20 °C to 85 °C as indicated in the legend. Five *IV* cycles per temperature.

The strongest temperature dependence can be observed for the HRS (lower current branch) at positive voltages. The other branches are substantially less temperature-dependent or even have an inverted temperature dependence in comparison with the positive-voltage HRS. As will be discussed later on, the currents are controlled mainly by Schottky emission at low and medium currents (with possible contributions from Poole-Frenkel emission) and by space-charge-limited conduction (SCLC) or trap-assisted tunneling (TAT) at the highest currents. Since the dominant current mechanisms change along the *IV* curves, so do the temperature dependences. Also, at high temperatures, the resistance of Nb:STO increases rapidly, almost exponentially, so while it should not limit the currents at room temperature and below, the increasing resistance can be expected to affect the currents at higher temperatures and interfere further with the temperature dependence of the film and barrier.

Fitting of electrical data

To support identifying the electronic transport mechanisms in the fabricated devices, different electronic conduction models were fitted to the measured data: the Schottky emission (SE) model, the space-charge-limited conduction (SCLC) model, the Poole-Frenkel (PF) emission model, and a trap-assisted tunneling (TAT) model were compared. In summary, based on electrical measurements, clear evidence is found for a thermionically controlled current injection into the switching insulator at low and intermediate currents with SCLC or TAT taking over at higher currents. As many thermionic processes have similar exponential dependences on the electric field (due to the Boltzmann approximation), a singular mechanism cannot be identified purely by electrical measurements and most likely, several processes occur in parallel. The detailed results are as follows.

For Schottky emission over a reverse-biased Schottky barrier (*53*) in a metal-insulator-semiconductor stack,

$$J_{\mathrm{SE}} = A^{*}T^{2}\exp\left[\frac{q}{k_{\mathrm{B}}T}\left(\sqrt{\frac{qV}{4\pi\epsilon_{\mathrm{op}}\epsilon_{0}}} - \mathit{\Phi}_{\mathrm{B}}\right)\right], \tag{S1}$$

where J_{SE} is the measured current density, $A^{*} = \lambda A_{0}$ is the effective Richardson constant, where $A_{0} = 1.2 \times 10^{6}\,\mathrm{Am^{-2}K^{-2}}$ and λ is typically of the order 0.5 (*71*), $T = 291\,\mathrm{K}$ is the measurement temperature, q the elemental charge, k_{B} the Boltzmann constant, V the applied voltage, ϵ_{op} the optical dielectric constant of the thin film, ϵ_{0} the vacuum permittivity, t_{ox} the thin film thickness, and $\mathit{\Phi}_{\mathrm{B}}$ the interface barrier height, which is to be calculated. This equation assumes full depletion of the dielectric so that the electric field across it can be approximated as $E = V/t_{\mathrm{ox}}$. Note that in the presence of partial filaments the effective thickness for establishing the electric field is smaller than the actual oxide film thickness. For the calculated barrier height values, the exact electric field will induce a shift of the values, since the effect will be similar for the barrier heights in the high and low resistance states, a qualitative comparison will not be affected by this. A comparison with the forward direction of a Schottky contact ($\propto V$ instead of $\propto \sqrt{V}$) revealed that the forward direction does not fit the measured values. From fits of (S1), the barrier height Φ_{B} can be calculated.

The only assumptions required for calculating $\mathit{\Phi}_{\mathrm{B}}$ from (S1) is that of λA_{0}, which on top of being the only input parameter appears in a logarithm when fitting the model so that its impact on the final result is minor. Here, $\lambda = 0.5$ was assumed as a typical value and A_{0} varies only with the effective mass m^{*} as $A_{0} = 4\pi q m^{*} k_{\mathrm{B}}^{2}/h^{3}$, where h is Planck's constant (*71*). The only other unknown in (S1) is ϵ_{op}, so its resulting value after fitting was used to gauge the physical reasonability of the fitting results. (This can be done independently from calculating $\mathit{\Phi}_{\mathrm{B}}$ as ϵ_{op} is calculated from the fitted slope, whereas $\mathit{\Phi}_{\mathrm{B}}$ is calculated from the y-intercept.) For all fits, ϵ_{op} was on the order of one, which is a very reasonable value for hafnium oxide, even in

the presence of dopants (*72*). From linear regression fits of $\ln(J)$ to $\sqrt{V}$ (see Fig. S18) for the ten devices in Fig. 1A), Φ_B calculated with (S1) and $m^* = 0.11 m_0$ (*73*) (m_0 electron rest mass) is $0.65 \pm 0.03\,\mathrm{eV}$ and $0.62 \pm 0.03\,\mathrm{eV}$ for the HRS in the positive and negative voltage directions, respectively, and $0.46 \pm 0.03\,\mathrm{eV}$ and $0.47 \pm 0.03\,\mathrm{eV}$ for the LRS in the positive and negative voltage ranges, respectively. When varying m^* between $0.11 m_0$ and $1 m_0$, these values increased by about 0.05-0.1 eV. The excellent fits, consistent for >50 devices, indicate that while again, no singular current mechanism can be identified reliably, Schottky emission seems to contribute a dominant portion of it. None of the other models yielded similarly close and consistent fits over the same current ranges.

To support the conclusion of Schottky emission as one of the dominant current transport mechanisms, besides calculating Φ_B by fitting (S1) as a function of voltage, it can also be fitted as a function of temperature. The results are presented in Fig. S19. The barrier heights calculated from these Arrhenius plots, however, are lower than the ones calculated from fitting SE as a function of voltage; $0.23 \pm 0.05\,\mathrm{eV}$ from temperature dependence vs. $0.65 \pm 0.03\,\mathrm{eV}$ from voltage dependence.

For SCLC, mathematical expressions vary depending on whether or not charge traps are involved while changing voltages (*55*). The 'extreme' cases for this are $I \propto V^2$ in the case

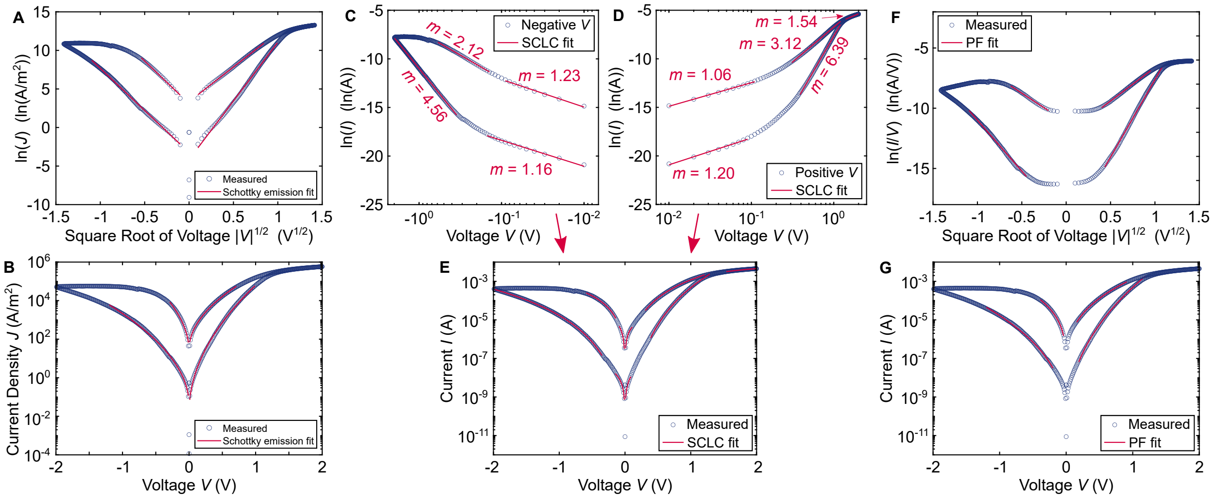

Figure S18: Different electronic transport models fitted to the measured *IV* curves. (**A**) Schottky emission: $\ln(J)$ vs. $\sqrt{V}$, (**B**) fitted model curve on top of measured *IV* curve. Space-charge-limited conduction (SCLC): $\ln(I)$ vs. V for (**C**) negative voltage range with inverted x-axis and (**D**) positive voltage range, (**E**) fitted model curve on top of measured *IV* curve. (**F**) Poole-Frenkel emission: $\ln(I/V)$ vs. $\sqrt{V}$, (**G**) fitted model curve on top of measured *IV* curve.

of trap-free materials and $I \propto \exp(V)$ in the case of traps uniformly distributed in energy and throughout the material. For different trap distributions in space and energy, virtually any exponent is possible for the voltage dependence. Thus, here, a linear fit was applied to $\ln(I)$ vs. $\ln(V)$ to reveal the exponent. Example fits are provided in Fig. S18C–E.

Linear fits in the double-logarithmic plots yield solid results in two different voltage ranges, with slopes of ≈ 1 for low, and >2 for larger voltages. In the literature, this is often interpreted

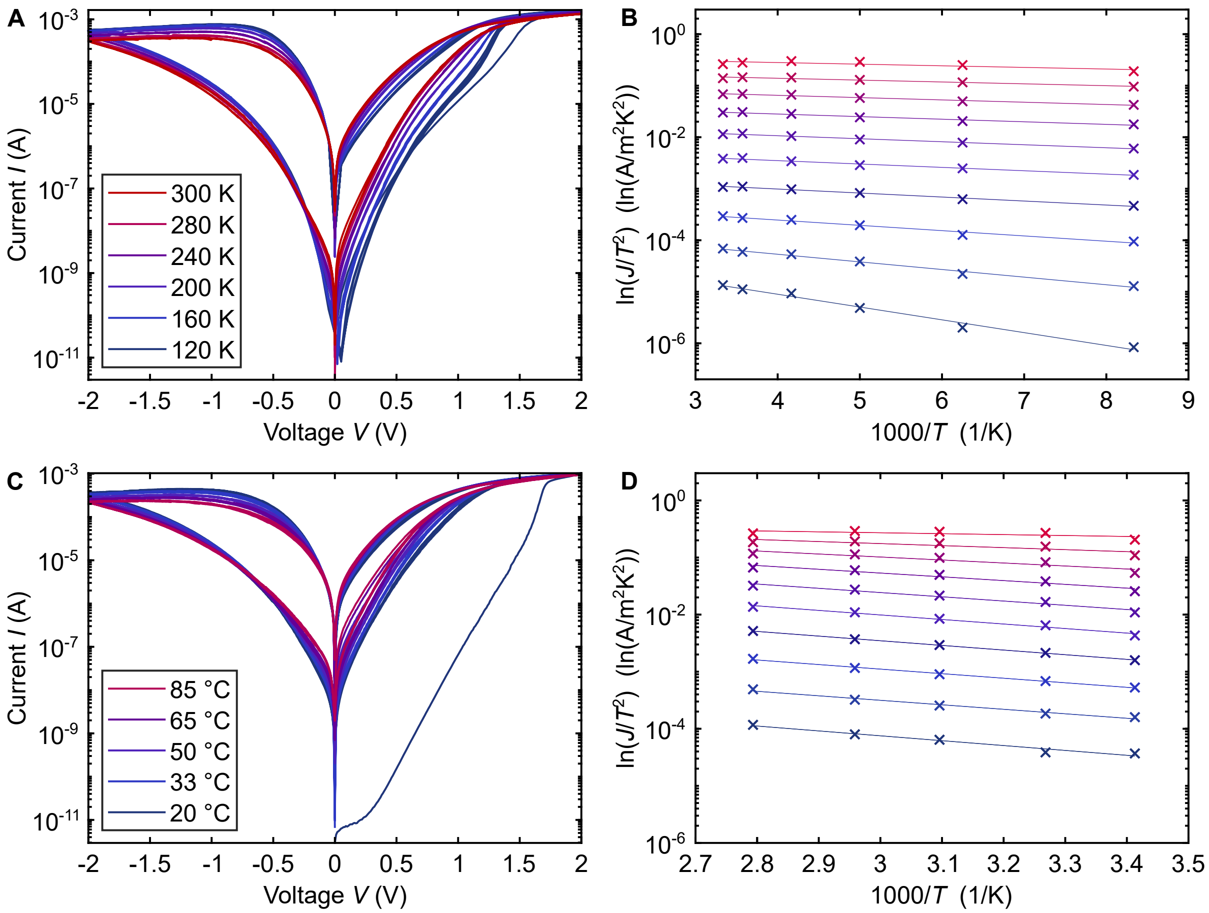

Figure S19: *IV* curves and Arrhenius plots for Ba:HfO$_x$ devices at temperatures from 120 K to 360 K. (**A**) & (**C**) Same plots as Fig. S17. In the HRS for positive voltages, a clear temperature dependence is visible. In the other branches of the *IV* curves, the dependence is much less pronounced and in may even change sign. Note especially the crossing of *IV* curves for the HRS in the negative voltage ranges. This will be discussed below. (**B**) & (**D**) The currents in the lower current branch at positive voltages, i.e. HRS, follows an Arrhenius-like temperature dependence. The points marked in (B) and (D) correspond to voltages from $0.1\,\mathrm{V}$ to $1\,\mathrm{V}$ in steps of $0.1\,\mathrm{V}$.

as a transition from ohmic transport (slope 1) due to thermionically generated charge carriers inside a material to SCLC in the presence of traps (slope >2). As the slope ≈ 1 only applies to a few data points at low voltages, we differ from this interpretation in concluding that at low voltages, SE is dominant, which then transitions to SCLC in the presence of traps at higher voltages, when large amounts of charge carriers are injected into the oxide. This is consistent with the ubiquity of traps in amorphous hafnium oxide. As an alternative, TAT dominates the high-current ranges, as discussed in the following.

The observed crossing of the *IV* curve in the negative-voltage HRS has been investigated in detail in e.g. (*74*) and provides further evidence as to the presence of different conduction mechanisms. In (*74*), TAT was identified as playing an important role in the current transport, so it is investigated here in the following and it turns out that this model can indeed describe the measured currents at the highest current levels. As TAT is temperature-independent, the inverted temperature difference of the measured *IV* curves in the negative-voltage after the *IV* crossing can then be explained by a phonon-limited bulk effect in the Ba:HfO$_x$ films.

The equation used for fitting the TAT model is (*54*)

$$J_{\mathrm{TAT}} = C \times \exp\left[-\frac{8\pi\sqrt{2qm^*}}{3hE}\,\Phi_{\mathrm{T}}^{3/2}\right], \tag{S2}$$

where C is a summary of constants, q the elemental charge, $m^* = 0.11m_0$ the effective mass in the oxide (with m_0 the electron rest mass) as before (*73*), h is Planck's constant, E the electric field approximated as $E = V/t_{\mathrm{ox}}$ with the applied voltage V and the oxide thickness t, and Φ_{T} is the depth in energy of the involved traps. As before, note that the effective thickness t_{ox} is lower in the presence of partial filaments, which effectively 'short-circuit' part of the film thickness. It is appropriate to use the highest applied voltages for the fitting as it is usually assumed that electrons tunnel into states into the oxide conduction band via traps, rather than

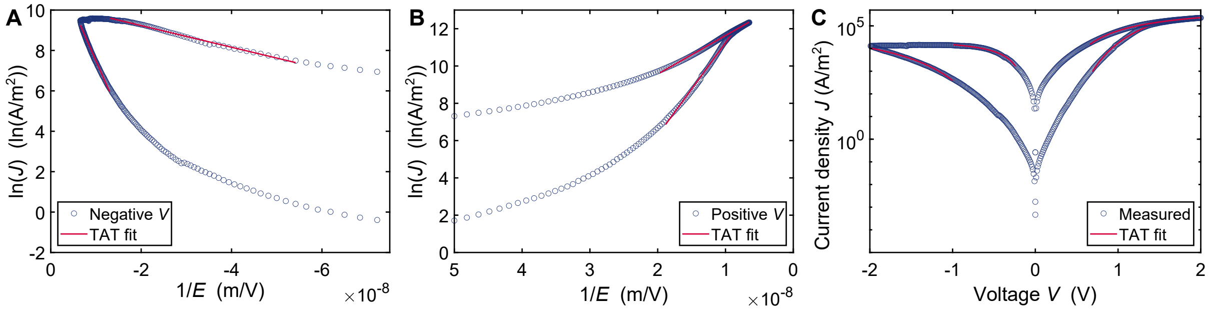

Figure S20: Fits of the trap-assisted tunneling (TAT) model to a measured *IV* curve. (**A**) & (**B**) Negative and positive voltage range, respectively. (**C**) Fitted data plotted on top of the originally measured data.

tunneling through the whole oxide thickness. (In this case, a hopping conduction mechanism would be more appropriate.) At low voltages, the conduction band edge is too high for electrons to tunnel into, i.e. there are no available states to tunnel into, and it requires a certain electric field to lower part of the conduction band below the energy level of the involved traps. The good fits with both the TAT and SCLC model at the highest currents suggest that they describe electron transport appropriately in these regions.

As with the thermionically assisted mechanisms before, the fits of (S2) to the high and low resistance state results in higher and lower energy levels for Φ_{T}, although for the TAT model, the interpretation for this difference is less straightforward than for a changing energy barrier height. One possible explanation would be the field-induced reversible creation and recombination of traps which add and remove defect levels at different Φ_{T}. As at this point, however, there is no further evidence for this, this is a conjecture which we will not take any further. Instead, we just want to point out that for all of the investigated models, the high and low resistance state clearly reveal a change of the height or depth of the involved energy levels.

Ultraviolet photoemission spectroscopy

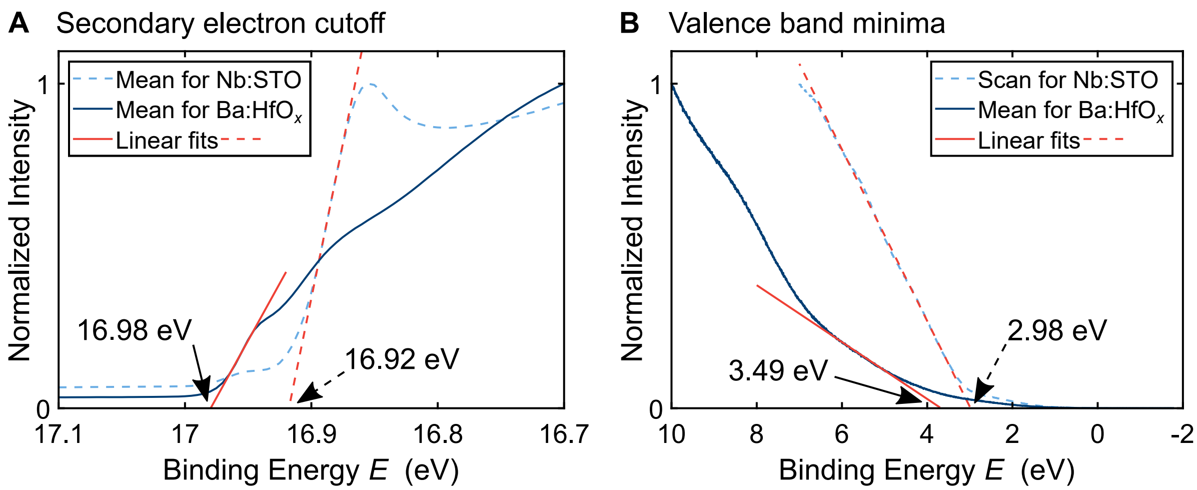

Figure S21: Ultraviolet photoelectron spectroscopy spectra. (**A**) Measurement of the vacuum offset between the substrate and the Ba:HfO$_x$ film and (**B**) the difference between the valence band minima (VBM). For Ba:HfO$_x$, the plotted spectra are the mean value of three measured scans. For (A), the Nb:STO spectrum is the average of two scans, and for (B), only one scan was carried out for the Nb:STO. The values provided in the figures are the extrapolated intersections with the x-axis.

REFERENCES AND NOTES

1. A. Chen, A review of emerging non-volatile memory (NVM) technologies and applications. *Solid State Electron.* **125**, 25–38 (2016).

2. S. K. W. Banerjee, A. Kashir, Hafnium oxide (HfO_2) – A multifunctional oxide: A review on the prospect and challenges of hafnium oxide in resistive switching and ferroelectric memories. *Small* **18**, 2107575 (2022).

3. S. D. S. Salahuddin, K. Ni, The era of hyper-scaling in electronics. *Nat. Electron.* **1**, 442–450 (2018).

4. S. Dirkmann, J. Kaiser, C. Wenger, T. Mussenbrock, Filament growth and resistive switching in hafnium oxide memristive devices. *ACS Appl. Mater. Interfaces* **10**, 14857–14868 (2018).

5. H. Y. Lee, Y. S. Chen, P. S. Chen, P. Y. Gu, Y. Y. Hsu, S. M. Wang, W. H. Liu, C. H. Tsai, S. Sheu, P. C. Chiang, W. P. Lin, C. H. Lin, W. S. Chen, F. T. Chen, C. H. Lien, M. J. Tsai, Evidence and solution of over-RESET problem for HfOX based resistive memory with sub-ns switching speed and high endurance, *Proceedings of the 2010 International Electron Devices Meeting*, San Francisco, CA, USA, 6–8 December 2010, pp. 19.7.1–19.7.4.

6. W. Kim, A. Hardtdegen, C. Rodenbucher, S. Menzel, D. J. Wouters, S. Hoffmann-Eifert, D. Buca, R. Waser, V. Rana, Forming-free metal-oxide ReRAM by oxygen ion implantation process, *Proceedings of the 2016 International Electron Devices Meeting (IEDM)*, San Francisco, CA, USA, 3–7 December 2017, pp. 4.4.1–4.4.4.

7. S. Petzold, A. Zintler, R. Eilhardt, E. Piros, N. Kaiser, S. U. Sharath, T. Vogel, M. Major, K. P. McKenna, L. Molina-Luna, L. Alff, Forming-free grain boundary engineered hafnium oxide resistive random access memory devices. *Adv. Electron. Mater.* **5**, 1900484 (2019).

8. M. S. Ram, K.-M. Persson, A. Irish, A. Jönsson, R. Timm, L.-E. Wernersson, High-density logic-in-memory devices using vertical indium arsenide nanowires on silicon. *Nat. Electron.* **4**, 914–920 (2021).

9. A. Fantini, L. Goux, R. Degraeve, D. J. Wouters, N. Raghavan, G. Kar, A. Belmonte, Y. Y. Chen, B. Govoreanu, M. Jurczak, Intrinsic switching variability in HfO2 RRAM, *Proceedings of the 2013 5th IEEE International Memory Workshop*, Monterey, CA, USA, 26–29 May 2013, pp. 30–33.

10. S. Ambrogio, S. Balatti, A. Cubeta, A. Calderoni, N. Ramaswamy, D. Ielmini, Statistical fluctuations in HfO_x resistive-switching memory: Part I-set/reset variability. *IEEE Trans. Electron Devices* **61**, 2912–2919 (2014).

11. A. Chen, Area and thickness scaling of forming voltage of resistive switching memories. *IEEE Electron Device Lett.* **35**, 57–59 (2014).

12. S. Kim, J. Chen, Y. C. Chen, M. H. Kim, H. Kim, M. W. Kwon, S. Hwang, M. Ismail, Y. Li, X. S. Miao, Y. F. Chang, B. G. Park, Neuronal dynamics in HfO_x/AlO_y-based homeothermic synaptic memristors with low-power and homogeneous resistive switching. *Nanoscale* **11**, 237–245 (2019).

13. W. Wu, H. Wu, B. Gao, N. Deng, S. Yu, H. Qian, Improving analog switching in HfO_x-based resistive memory with a thermal enhanced layer. *IEEE Electron Device Lett.* **38**, 1019–1022 (2017).

14. Q. Luo, X. Zhang, Y. Hu, T. Gong, X. Xu, P. Yuan, H. Ma, D. Dong, H. Lv, S. Long, Q. Liu, M. Liu, Self-rectifying and forming-free resistive-switching device for embedded memory application. *IEEE Electron Device Lett.* **39**, 664–667 (2018).

15. J. J. Ryu, K. Jeon, S. Yeo, G. Lee, C. Kim, G. H. Kim, Fully "erase-free" multi-bit operation in HfO_2-based resistive switching device, *ACS Appl. Mater. Interfaces* **11**, 8234–8241 (2019).

16. S. Bagdzevicius, K. Maas, M. Boudard, M. Burriel, Interface-type resistive switching in perovskite materials. *J. Electroceram.* **39**, 157–184 (2017).

17. B. J. Choi, A. C. Torrezan, K. J. Norris, F. Miao, J. P. Strachan, M.-X. Zhang, D. A. A. Ohlberg, N. P. Kobayashi, J. J. Yang, R. S. Williams, Electrical performance and scalability of Pt dispersed SiO_2 nanometallic resistance switch. *Nano Lett.* **13**, 3213–3217 (2013).

18. B. Govoreanu, L. Di Piazza, J. Ma, T. Conard, A. Vanleenhove, A. Belmonte, D. Radisic, M. Popovici, A. Velea, A. Redolfi, O. Richard, S. Clima, C. Adelmann, H. Bender, M. Jurczak, Advanced a-VMCO resistive switching memory through inner interface engineering with wide (>10^2) on/off window, tunable μA-range switching current and excellent variability, *Proceedings of the 2016 IEEE Symposium on VLSI Technology*, Honolulu, HI, USA, 14–16 June 2016, pp. 1–2.

19. M. Kim, M. A. Rehman, D. Lee, Y. Wang, D.-H. Lim, M. F. Khan, H. Choi, Q. Y. Shao, J. Suh, H.-S. Lee, H.-H. Park, Filamentary and interface-type memristors based on tantalum oxide for energy-efficient neuromorphic hardware. *ACS Appl. Mater. Interfaces* **14**, 44561–44571 (2022).

20. M. Lederer, D. Lehninger, T. Ali, T. Kämpfe, Review on the microstructure of ferroelectric hafnium oxides. *Phys. Status Solidi Rapid Res. Lett.* **16**, 2200168 (2022).

21. M. Lanza, H. S. P. Wong, E. Pop, D. Ielmini, D. Strukov, B. C. Regan, L. Larcher, M. A. Villena, J. J. Yang, L. Goux, A. Belmonte, Y. Yang, F. M. Puglisi, J. Kang, B. Magyari-Köpe, E. Y. A. Kenyon, M. Buckwell, A. Mehonic, A. Shluger, H. Li, T. H. Hou, B. Hudec, D. Akinwande, R. Ge, S. Ambrogio, J. B. Roldan, E. Miranda, J. S. Ne, K. L. Pey, X. Wu, N. Raghavan, E. Wu, W. D. Lu, G. Navarro, W. Zhang, H. Wu, R. Li, A. Holleitner, U. Wurstbauer, M. C. Lemme, M. Liu, S. Long, Q. Liu, H. Lv, A. Padovani, P. Pavan, I. Valov, X. Jing, T. Han, K. Zhu, S. Chen, F. Hui, Y. Shi, Recommended methods to study resistive switching devices. *Adv. Electron. Mater.* **5**, 1800143 (2019).

22. B. Johnson, J. L. Jones, Structures, phase equilibria, and properties of HfO2, in *Ferroelectricity in Doped Hafnium Oxide: Materials, Properties and Devices* (Woodhead Publishing Series in Electronic and Optical Materials, 2019), pp. 25–45.

23. S. Cho, C. Yun, S. Tappertzhofen, A. Kursumovic, S. Lee, P. Lu, Q. Jia, M. Fan, J. Jian, H. Wang, S. Hofmann, J. L. MacManus-Driscoll, Self-assembled oxide films with tailored nanoscale ionic and electronic channels for controlled resistive switching. *Nat. Commun.* **7**, 12373 (2016).

24. X. Gu, D. Lubyshev, J. Batzel, J. M. Fastenau, W. K. Liu, R. Pelzel, J. F. Magana, Q. Ma, L. P. Wang, P. Zhang, V. R. Rao, Commercial molecular beam epitaxy production of high quality $SrTiO_3$ on large diameter Si substrates. *J. Vac. Sci. Technol. B* **27**, 1195–1199 (2009).

25. E. Mikheev, B. D. Hoskins, D. B. Strukov, S. Stemmer, Resistive switching and its suppression in Pt/Nb:$SrTiO_3$ junctions. *Nat. Commun.* **5**, 3990 (2014).

26. S. Kunwar, C. B. Somodi, R. A. Lalk, B. X. Rutherford, Z. Corey, P. Roy, D. Zhang, M. Hellenbrand, M. Xiao, J. L. MacManus-Driscoll, Q. Jia, H. Wang, J. J. Yang, W. Nie, A. Chen, Protons: Critical species for resistive switching in interface-type memristors. *Adv. Electron. Mater.* **9**, 2200816 (2023).

27. E. R. Hsieh, K. T. Chen, P. Y. Chen, S. S. Wong, S. S. Chung, A forming-free HfO_2-/HfON-based resistive-gate metal-oxide-semiconductor field-effect-transistor (RG-MOSFET) nonvolatile memory with 3-bit-per-cell storage capability. *IEEE Trans. Electron Devices* **68**, 2699–2704 (2021).

28. S. Schlachter, B. Drake, Introducing micron DDR5 SDRAM: More than a generational update, *White paper*, Micron Technology Inc. (2019).

29. A. Wadsworth, *The Parametric Measurement Handbook* (Keysight Technologies, 2017), chap. 5.

30. S. Yu, Y. Wu, R. Jeyasingh, D. Kuzum, H. S. P. Wong, An electronic synapse device based on metal oxide resistive switching memory for neuromorphic computation. *IEEE Trans. Electron Devices* **58**, 2729–2737 (2011).

31. V. Pandey, Origin of the Curie-von Schweidler law and the fractional capacitor from time-varying capacitance. *J. Power Sources* **532**, 231309 (2022).

32. Z. Wang, H. Wu, G. W. Burr, C. S. Hwang, K. L. Wang, Q. Xia, J. J. Yang, Resistive switching materials for information processing. *Nat. Rev. Mater.* **5**, 173–195 (2020).

33. L. F. Abbott, S. B. Nelson, Synaptic plasticity: Taming the beast. *Nat. Neurosci.* **3**, 1178–1183 (2000).

34. M. Henning, Theoretical models of synaptic short term plasticity. *Front. Comput. Neurosci.* **7**, 45 (2013).

35. I. Valov, Interfacial interactions and their impact on redox-based resistive switching memories (ReRAMs). *Semicond. Sci. Technol.* **32**, 093006 (2017).

36. Y. Munakata, J. Pfaffly, Hebbian learning and development. *Dev. Sci.* **7**, 141–148 (2004).

37. A. L. Hodgkin, A. F. Huxley, A quantitative description of membrane current and its application to conduction and excitation in nerve. *J. Physiol.* **117**, 500–544 (1952).

38. W. B. Levy, O. Steward, Temporal contiguity requirements for long-term associative potentiation/depression in the hippocampus. *Neuroscience* **8**, 791–797 (1983).

39. B. Max, M. Hoffmann, H. Mulaosmanovic, S. Slesazeck, T. Mikolajick, Hafnia-based double-layer ferroelectric tunnel junctions as artificial synapses for neuromorphic computing. *ACS Appl. Electron. Mater.* **2**, 4023–4033 (2020).

40. M. Koleva, P. Atanasov, R. Tomov, O. Vankov, C. Matin, C. Ristoscu, I. Mihailescu, D. Iorgov, S. Angelova, C. Ghelev, N. Mihailov, Pulsed laser deposition of barium hexaferrite ($BaFe_{12}O_{19}$) thin films. *Appl. Surf. Sci.* **154**, 485–491 (2000).

41. H. Bae, Y. Lee, K. J. Kim, G. M. Choi, Effects of fabrication conditions on the crystallinity, barium deficiency, and conductivity of $BaZr_{0.8}Y_{0.2}O_{3-\delta}$ films grown by pulsed laser deposition. *Fuel Cells* **15**, 408–415 (2015).

42. B. Bakhit, D. Primetzhofer, E. Pitthan, M. A. Sortica, E. Ntemou, J. Rosen, L. Hultman, I. Petrov, G. Greczynski, Systematic compositional analysis of sputter-deposited boron-containing thin films. *J. Vac. Sci. Technol. A* **39**, 063408 (2021).

43. R. C. Smith, T. Ma, N. Hoilien, L. Y. Tsung, M. J. Bevan, L. Colombo, J. Roberts, S. A. Campbell, W. L. Gladfelter, Chemical vapour deposition of the oxides of titanium, zirconium and hafnium for use as high-*k* materials in microelectronic devices. A carbon-free precursor for the synthesis of hafnium dioxide. *Adv. Funct. Mater.* **10**, 105–114 (2000).

44. A. S. Foster, F. L. Gejo, A. L. Shluger, R. M. Nieminen, Vacancy and interstitial defects in hafnia. *Phys. Rev. B Condens. Matter Mater. Phys.* **65**, 1741171–17411713 (2002).

45. C. Tang, R. Ramprasad, Point defect chemistry in amorphous HfO_2: Density functional theory calculations. *Phys. Rev. B Condens. Matter Mater. Phys.* **81**, 161201 (2010).

46. M. G. Blamire, J. L. MacManus-Driscoll, N. D. Mathur, Z. H. Barber, The materials science of functional oxide thin films. *Adv. Mater.* **21**, 3827–3839 (2009).

47. S. M. Alay-E-Abbas, A. Shaukat, First-principles study of thermodynamic stability and the electronic properties of intrinsic vacancy defects in barium hafnate. *J. Phys. Condens. Matter* **26**, 435501 (2014).

48. T. V. Perevalov, V. A. Gritsenko, S. B. Erenburg, A. M. Badalyan, H. Wong, C. W. Kim, Atomic and electronic structure of amorphous and crystalline hafnium oxide: X-ray photoelectron spectroscopy and density functional calculations. *J. Appl. Phys.* **101**, 053704 (2007).

49. G. Greczynski, L. Hultman, Towards reliable x-ray photoelectron spectroscopy: Sputter-damage effects in transition metal borides, carbides, nitrides, and oxides. *Appl. Surf. Sci.* **542**, 148599 (2021).

50. K. Xiong, J. Robertson, Point defects in HfO_2 high K gate oxide. *Microelectron. Eng.* **80**, 408–411 (2005).

51. E. Hildebrandt, J. Kurian, M. M. Müller, T. Schroeder, H. J. Kleebe, L. Alff, Controlled oxygen vacancy induced *p*-type conductivity in HfO_{2-x} thin films. *Appl. Phys. Lett.* **99**, 112902 (2011).

52. A. Sawa, Resistive switching in transition metal oxides. *Mater. Today* **11**, 28–36 (2008).

53. S. M. Sze, K. K. Ng, *Physics of Semiconductor Devices* (John Wiley & Sons Inc., 2007), chap. 4.

54. M. P. Houng, Y. H. Wang, W. J. Chang, Current transport mechanism in trapped oxides: A generalized trap-assisted tunneling model. *J. Appl. Phys.* **86**, 1488–1491 (1999).

55. M. A. Lampert, Simplified theory of space-charge-limited currents in an insulator with traps. *Phys. Rev.* **103**, 1648–1656 (1956).

56. G. D. Derry, M. E. Kern, E. H. Worth, Recommended values of clean metal surface work functions. *J. Vac. Sci. Technol. A* **33**, 060801 (2015).

57. C. Baeumer, C. Schmitz, A. Marchewka, D. N. Mueller, R. Valenta, J. Hackl, N. Raab, S. P. Rogers, M. I. Khan, S. Nemsak, M. Shim, S. Menzel, C. M. Schneider, R. Waser, R. Dittmann, Quantifying redox-induced schottky barrier variations in memristive devices via *in operando* spectromicroscopy with graphene electrodes. *Nat. Commun.* **7**, 12398 (2016).

58. M. Schie, M. P. Müller, M. Salinga, R. Waser, R. A. D. Souza, Ion migration in crystalline and amorphous HfO_x. *J. Chem. Phys.* **146**, 094508 (2017).

59. J. Strand, P. L. Torraca, A. Padovani, L. Larcher, A. L. Shluger, Dielectric breakdown in HfO_2 dielectrics: Using multiscale modeling to identify the critical physical processes involved in oxide degradation. *J. Appl. Phys.* **131**, 234501 (2022).

60. R. Waser, R. Dittmann, C. Staikov, K. Szot, Redox-based resistive switching memories nanoionic mechanisms, prospects, and challenges. *Adv. Mater.* **21**, 2632–2663 (2009).

61. S. Stille, C. Lenser, R. Dittmann, A. Koehl, I. Krug, R. Muenstermann, J. Perlich, C. M. Schneider, U. Klemradt, R. Waser, Detection of filament formation in forming-free resistive switching $SrTiO_3$ devices with Ti top electrodes. *Appl. Phys. Lett.* **100**, 223503 (2012).

62. R. Muenstermann, T. Menke, R. Dittmann, M. Shaobo, C. L. Jia, D. Park, J. Mayer, Correlation between growth kinetics and nanoscale resistive switching properties of $SrTiO_3$ thin films. *J. Appl. Phys.* **108**, 124504 (2010).

63. T. V. Perevalov, V. S. Aliev, V. A. Gritsenko, A. A. Saraev, V. V. Kaichev, Electronic structure of oxygen vacancies in hafnium oxide. *Microelectron. Eng.* **109**, 21–23 (2013).

64. L. Pintilie, M. Alexe, Metal-ferroelectric-metal heterostructures with schottky contacts. I. influence of the ferroelectric properties. *J. Appl. Phys.* **98**, 124103 (2005).

65. A. Toriumi, K. Kita, K. Tomida, Y. Yamamoto, Doped HfO_2 for higher-k dielectrics. *ECS Trans.* **1**, 185–197 (2006).

66. P. K. Hurley, K. Cherkaoui, E. O'Connor, M. C. Lemme, H. D. B. Gottlob, M. Schmidt, S. Hall, Y. Lu, O. Buiu, B. Raeissi, J. Piscator, O. Engstrom, S. B. Newcomb, Interface defects in HfO_2, $LaSiO_x$, and Gd_2O_3 high-*k*/metal-gate structures on silicon. *J. Electrochem. Soc.* **155**, G13 (2008).

67. M. Mayer, W. Eckstein, H. Langhuth, F. Schiettekatte, U. V. Toussaint, Computer simulation of ion beam analysis: Possibilities and limitations. *Nucl. Instrum. Methods Phys. Res., Sect. B* **269**, 3006–3013 (2011).

68. G. Greczynski, L. Hultman, X-ray photoelectron spectroscopy: Towards reliable binding energy referencing, *Prog. Mater. Sci.* **107**, 100591 (2020).

69. D. A. Shirley, High-resolution x-ray photoemission spectrum of the valence bands of gold. *Phys. Rev. B* **5**, 4709–4714 (1972).

70. M. Minohara, I. Ohkubo, H. Kumigashira, M. Oshima, Band diagrams of spin tunneling junctions $La_{0.6}Sr_{0.4}MnO_3$/Nb:SrTiO and $SrRuO_3$/Nb:$SrTiO_3$ determined by in situ photoemission spectroscopy. *Appl. Phys. Lett.* **90**, 132123 (2007).

71. C. R. Crowell, The Richardson constant for thermionic emission in schottky barrier diodes. *Solid State Electron.* **8**, 395–399 (1965).

72. H. Dou, N. Strkalj, Y. Zhang, J. L. MacManus-Driscoll, Q. Jia, H. Wang, Optical dielectric properties of HfO_2-based films. *J. Vac. Sci. Technol. A* **40**, 033412 (2022).

73. S. Monaghan, P. K. Hurley, K. Cherkaoui, M. A. Negara, A. Schenk, Determination of electron effective mass and electron affinity in HfO_2 using MOS and MOSFET structures. *Solid State Electron.* **53**, 438–444 (2009).

74. C. Funck, A. Marchewka, C. Bäumer, P. C. Schmidt, P. Müller, R. Dittmann, M. Martin, R. Waser, S. Menzel, A theoretical and experimental view on the temperature dependence of the electronic conduction through a schottky barrier in a resistively switching $SrTiO_3$-based memory cell. *Adv. Electron. Mater.* **4**, 1800062 (2018).
